# The Effects of Combined Physical and Cognitive Training on Inhibitory Control: A Systematic Review and Meta-Analysis

**DOI:** 10.1016/j.neubiorev.2021.07.008

**Published:** 2021-07-10

**Authors:** Sakshi Dhir, Wei-Peng Teo, Samuel R. Chamberlain, Kaelasha Tyler, Murat Yücel, Rebecca A. Segrave

**Affiliations:** 1 BrainPark, Turner Institute for Brain and Mental Health, School of Psychological Sciences and Monash Biomedical Imaging Facility, Monash University, Melbourne, Victoria, Australia; 2 Physical Education and Sports Science Academic Group, National Institute of Education, Nanyang Technological University, Singapore; 3 Institute for Physical Activity and Nutrition, Deakin University, Melbourne, Australia; 4 Department of Psychiatry, Faculty of Medicine, University of Southampton, UK; & Southern Health NHS Foundation Trust, UK

## Introduction

The ability to exert control over behavioural urges or unhelpful thoughts plays a key role in mental health and wellbeing (Aron, 2007; Aron, Fletcher, Bullmore, Sahakian, Robbins, 2013; Bari & Robbins, 2013; Barkley, 1997; Chamberlain, Blackwell, Fineberg, Robbins & Sahakian, 2005). Impairment in this ability, inhibitory control, is also a core feature of many neurological conditions and mental illnesses characterised by intrusive thoughts and behavioural impulsivity, such as obsessive compulsive disorder (OCD) (Berlin & Lee, 2018; Chamberlain et al., 2005; Lipszyc & Schachar, 2010), substance and behavioural addictions (Lubman, Yücel, & Pantelis, 2004; Luijten et al., 2014; Smith, Mattick, Jamadar & Iredale, 2014; Yücel, Lubman, Solowij & Brewer, 2007; Yucel & Lubman, 2007), Parkinson’s disease (Obeso et al., 2011), and attention deficit hyperactivity disorder (ADHD) (Lipszyc & Schachar, 2010). Currently there are no interventions that effectively and directly enhance inhibitory control.

However, over the last decade there has been a rapid increase in evidence demonstrating that regular patterns of human behaviour have major effects on neurocognitive systems (Levin, Netz & Ziv, 2021; Stillman et al., 2016). With this insight has come growing interest in the capacity of behavioural interventions, such as cognitive training and physical training, to potentially improve inhibitory control. As these approaches are generally accessible, acceptable, scalable, and safe, they have significant potential to help a wide range of individuals enhance their inhibitory control, and consequently their mental wellbeing and quality of life (Diepeveen et al., 2013; Devereux-Fitzgerald, Powell, Dewhurst, & French, 2016).

### Cognitive Training

To date, cognitive training is the only well-known behavioural intervention that has been designed to specifically target and modify inhibitory control systems across disorders. Cognitive training involves repeated engagement in tasks that target cognitive skills and their associated neurocircuits. When targeting inhibitory control (i.e., inhibitory control training), tasks that exercise inhibitory control, such as the Stroop or Stop Signal task, are engaged in regularly over a period of time (from hours to several months). The goal of training is to activate and over time ‘strengthen’ the neural systems (e.g., right inferior frontal gyrus [rIGF] and its connections) that underpin inhibitory control via repeated practice, and in doing so improve inhibitory control (Haier et al, 1992; Hempel et al, 2004; Olesen et al., 2004; Olesen & Westerberg & Klingberg, 2004). Ultimately, the aim is to transfer benefits of training (e.g., resisting the urge to respond on a computer) to unhelpful behaviours (e.g., resisting the urge to repeatedly wash hands). However, while there is modest evidence for neural adaptations following inhibitory control training (Kuhn et al. 2017), the evidence for transfer of training effects to behaviour is not compelling. In particular, Sala et al.’s (2019) second order meta-analysis (a meta-analysis of meta-analyses) found no transfer of cognitive improvements on any behaviour beyond the trained task, regardless of type of population or training paradigm. The reviews of literature to date show limited evidence for the efficacy of inhibition control training on behaviour beyond the task practiced, and compounding this is also significant heterogeneity across training protocols (e.g., training tasks, dose, populations) (Cortese et al., 2015; Sonuga-Barke, Brandeis, Holtmann & Cortese, 2014). Moreover, much of the literature does not examine differences in training protocols or participant characteristics targeted to clarify the most effective training parameters and the associated effects. Thus, the current evidence indicates a lack of gold-standard inhibitory control training parameters and poor efficacy of benefits transferring to real-world inhibitory control related behaviours.

### Physical Training

Unlike cognitive training, the impacts of physical training, i.e., physical exercise, on brain health and cognition are broad and non-specific. Animal models demonstrate that physical training can induce a cascade of neuroplastic processes. For example, running in mice has been linked to increases in neurogenesis (Kempermann, Kuhn, & Gage, 1998; Kempermann, Kuhn, & Gage, 1997), neuronal survival into old age (Van Praag, Christie, Sejnowski & Gage, 1999; Van Praag, Kempermann & Gage, 1999), and synaptogenesis (Van Praag, Christie et al., 1999; Farmer et al. 2004; O’Callaghan, Ohle, R & Kelly, 2007). Throughout the animal literature these changes are most commonly shown within the hippocampus (Brown et al., 2003; Van Praag et al., 1999a; Van Praag et al., 1999b) and dendate gyrus (Van Praag, 2008), however neuroplastic changes post physical training have also been shown across frontal (Mandyam et al., 2007) and motor cortices (Ehninger & Kempermann, 2003). Importantly, neuromodulator effects of physical training have been replicated in older animals experiencing age-related decline (Kronenberg et al., 2006). Additionally, animal models have robustly demonstrated an increased release of growth factors (e.g., BDNF expression (Farmer et al. 2004) and increase IFG-1 uptake (Carro, Trejo, Busiguina & Torres-Aleman, 2001)) and modulations across neurotransmitter systems (e.g., glutamate (Farmer et al. 2004), serotonin, noradrenaline and acetylcholine (Lista & Sorrentino, 2010), and dopamine (Fordyce & Farrar, 1991)). Collectively, pre-clinical research provides multi-modal evidence that physical training can affect numerous aspects of neuroplasticity.

Whilst human research is not able to utilise invasive methods and as such lacks some of the precision and control of animal-derived evidence, a large literature has also reported robust neuroplastic change in response to physical training in humans (Kandola, Hendrikse & Yücel, 2016; Stillman et al., 2016). Further, physical training has been shown to improve many aspects of neurocognition in humans, including inhibitory control (Xue, Yang & Huang, 2019). For instance, the pre-supplementary motor area (pre-SMA) plays a key role in inhibitory control of behaviour (e.g., compulsive hand washing or impulsively reaching for another alcoholic drink) (Arantes et al., 2017; Bari & Robbins, 2013; Rector, Richter, Lerman & Regev, 2015). Thus, studies demonstrating physical training induced GABA- modulations within the motor cortex provide evidence for physical training’s benefits on mechanisms of inhibitory control (Castro-Alamancos & Connors, 1996; Coxon et al., 2018; Mooney et al., 2016; Singh, Duncan, Neva & Staines, 2014; Smith et al., 2014; Stavrinos & Coxon, 2017; Rioult-Pedotti, Friedman & Donoghue, 2000). Similarly, increased default mode network (DMN) activity is associated with poorer inhibitory control (Congdon et al., 2010), thus physical training’s decrease of DMN may have an indirect benefit on inhibitory control mechanisms (Boraxbekk, Salami, Wåhlin & Nyberg, 2016; Li et al., 2017). Additionally, physical training literature has demonstrated structural and functional physical training induced benefits for memory (Wanner, Cheng & Steib, 2020; Wilckens et al., 2020) and higher-level executive functions such as inhibition control (Verburgh, Königs, Scherder & Oosterlaan, 2014; Xue, Yang & Huang, 2019). For example, regular physical training is associated with increased grey matter volume in the prefrontal cortices (Erickson, Leckie, & Weinstein, 2014; Den Ouden et al., 2018), which support a range of executive functions including inhibitory control (Aron, Robbins & Poldrack, 2014; Bari & Robins, 2013; Bird & Burgess, 2008; Kim, Choi & Chung, 2016; Kim & Sung, 2017; Park & Kim, 2017; Swick, Ashley & Turken, 2011. However, a recent meta-analysis of the effects of physical training on brain volume in older adults (Gogniat, Robinson, & Miller, 2021) excluded the likelihood of brain volume to be the mechanism driving the relationship between physical training and cognitive benefits. These mixed findings reflect the partially unknown nature of the mechanisms behind the physical training and cognition link. Nonetheless, whilst the mechanisms underlying the neuroplastic and neuroprotective effects of exercise on cognition and the brain are not fully understood, there is convergent evidence from animal and human research showing that physical training can have a powerful effect on both.

The majority of the physical training and cognition research has focussed on memory modification in older people, with comparatively limited research investigating effects on inhibitory control. Nonetheless, a small number of studies have reported physical training induced inhibitory control improvements via electrophysiology changes (shorter P300 latencies and greater amplitudes indicative of faster inhibitory control processes) (Hillman et al., 2014), shorter Stroop interference reaction time (Hyodo et al., 2016), increased activation across brain regions involved in inhibitory control (Chaddock-Heyman et al., 2013; Colcombe et al., 2004; Krafft et al., 2014), and structural brain changes subserving enhanced inhibitory control (Verstynen et al., 2012; Weinstein et al., 2012). These studies have positioned physical training as a potential candidate for remediating inhibitory control impairments.

Across this literature, different physical training parameters have been associated with different cognitive outcomes for different individuals. For example, large scale meta-analyses show small (Chang, Labban, Gapin & Etnier, 2012; Etnier et al., 1997, Ludyga et al., 2020) to moderate (Heyn, Abreu, & Ottenbacher, 2004; Smith et al., 2010) benefits on general cognitive functions. However, Gates, Singh, Sachdev & Valenzuela’s (2013) meta-analysis of randomised controlled trials investigating physical training for older adults found an almost negligible effect (EF: 0.17). A key reason for this variability across outcomes is the heterogeneity in physical training parameters (e.g., exercise intensity, dose and modality) and participant groups investigated. For example, Gates et al. (2013) investigated MCI; Heyn et al. (2004) investigated cognitive impairment and dementia; and Chang et al. (2012), Etnier et al. (1997), Ludyga et al. (2020) and Smith et al. (2010) investigated adults without any specific inclusion criteria. Physical training interventions have shown different outcomes when delivered to individuals with different demographics (e.g., age) and different characteristics (e.g., AHDH, OCD, addictions, poor health) (Ludyga et al., 2020; Stillman et al., 2020). Similarly, training parameters (i.e., exercise intensity, dose and modality) also have a critical impact on the effect of physical training on cognitive outcomes, just as they do on physical outcomes. Physical training intensity has shown a particularly pronounced impact on cognitive outcomes, with some reviews finding moderate intensity to have a more potent impact (Chang et al., 2012) and others finding that acute high intensity is superior (Fiorelli et al., 2019; Tsukamoto et al., 2016). Thus, there is a clear role of physical training intensity on neural changes and associated cognitive outcomes, and widespread inter-individual differences in effects. However, these moderators of physical training effectiveness have not been clarified or considered across much of the research conducted to date, despite their critical impact on outcomes.

### Combined Physical and Cognitive Training

The combination of physical and cognitive training may have synergistic effects on cognition, including inhibitory control. One influential theory is Raichen & Alexander’s (2017) ‘Adaptive Capacity Model’ (ACM), which takes an evolutionary neuroscience perspective based on the observation that humans evolved to engage in both physical and cognitive activity simultaneously in order to survive whilst living through a hunting and gathering-type lifestyle. ACM proposes that many of our neurobiological and cognitive systems were honed specifically to respond to these combined cognitive and physical challenges. For example, hunting involved demands on aspects of memory and spatial navigation, combined with physical movement to search for food in complex environments. As such, ACM suggests that this physical exercise during complex cognitive activity potentially resulted in enhanced neuroplastic benefits, such that survival of exercise induced neurons may be enhanced by the addition of cognitive challenges. These benefits from combined physical and cognitive activity may have safeguarded against the inactivity-induced cognitive decline now associated with our sedentary lifestyle. Thus, simultaneous engaging in physical and cognitive activity may result in benefits for important cognitive facets, such as inhibition control.

**Figure 1:**
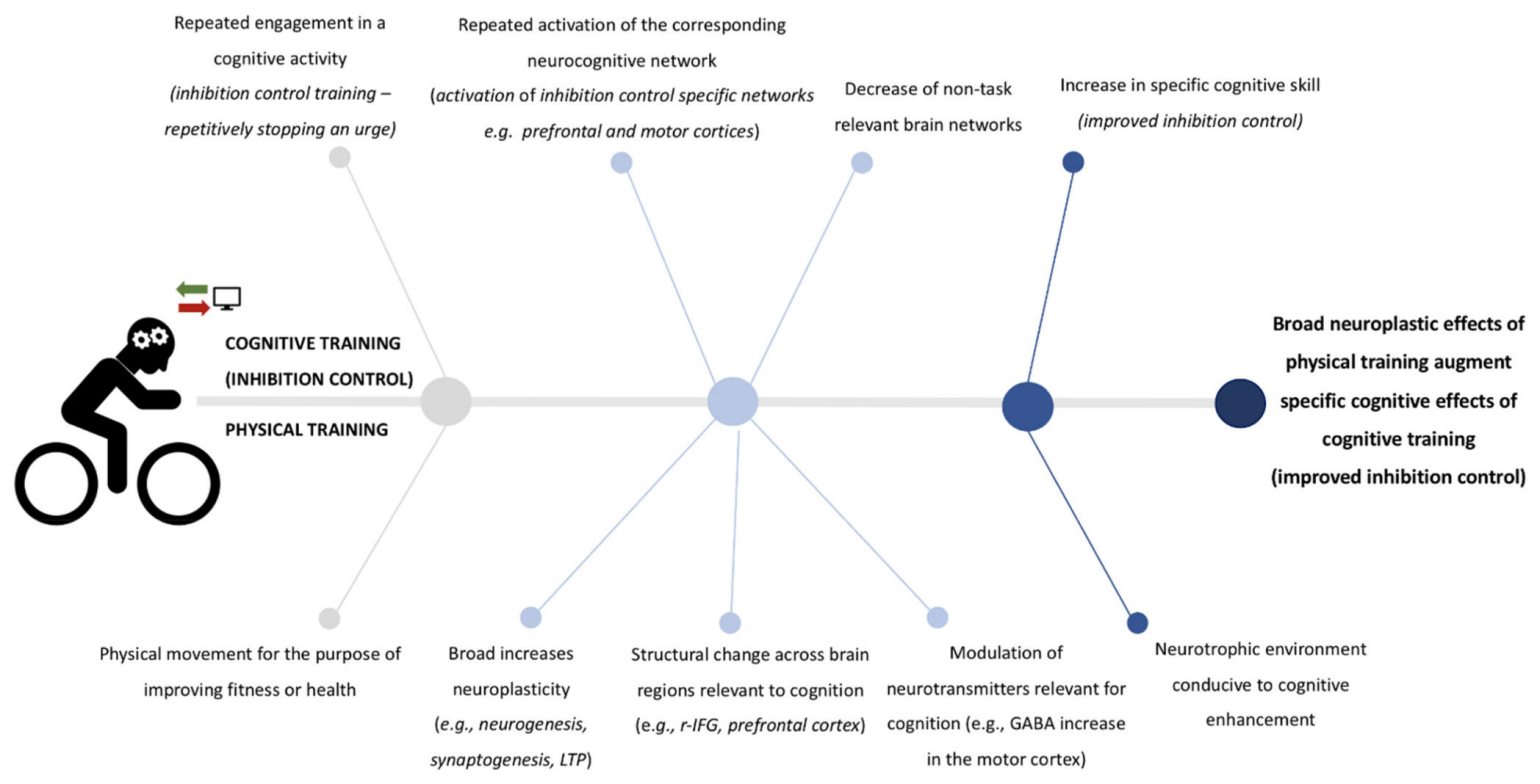
Potential mechanisms behind the neurocognitive effects of combining physical and cognitive training to strengthen inhibitory control. Note: this figure requires publishing in colour

Moreover, as noted above, physical training has broad effects on the brain and cognition, including enhancing neuroplasticity, whereas cognitive training is designed to have specific effects and enhance functioning in the discrete neurocognitive domain being targeted. Thus, from a theoretical perspective, neuroplasticity induced by physical training could create a neurotrophic environment whereby the brain is more receptive to the specific effects of cognitive training, ultimately resulting in greater likelihood of long term and transferable improvements. See Figure 1 for this process exemplified for inhibitory control. Studies in mice have partially supported this rationale, where wheel running in enriched environments (Fabel et al., 2009) or physical training involving cognitive challenges (Motta-Teixeira et al., 2016) have been associated with significantly increased neurogenesis compared to tradition wheel running. Similar to the model outlined in Figure 1, Hötting & Röder (2013) comprehensively reviewed the literature investigating physical training’s impact on cognition, with a focus on both animal and human models, and proposed that the induction of neurogenesis through physical training alone may be insufficient to induce long lasting structural changes in the brain for functional benefit (see Gogniat et al. 2021). However, exercise-induced new neurons are more likely to survive and result in sustained cognitive enhancement when physical training is functionally integrated via the addition of cognitive activity. Hotting and Röder (2013) echo the hypothesis that physical training may prime the neuroplastic brain to augment cognitive training’s specific benefits. Thus, combining physical and cognitive training may produce greater effects than either modality alone and, consequently result in improved lasting benefits for inhibitory control.

In recognition of the above, a rapidly growing number of research investigations are testing the efficacy of combined physical and cognitive training paradigms for cognitive enhancement, with a particular focus on inhibitory control. The literature has shown benefits of combined training, often compared to exercise alone, on inhibitory control cognitive outcomes in ageing populations (Anderson-Hanley et al., 2012; Barcelos et al., 2015; Kuhn et al., 2017; Shatil, 2013), in individuals with mild cognitive impairment (Sacco et al., 2016), children (Staiano, Abraham & Calvert, 2012), and in healthy adult populations (Ward et al., 2017). A recent meta-analysis found that one of the most potent moderators of physical training’s benefits for cognition was the use of coordinative exercises (i.e., involving cognitive challenges), compared to traditional types of physical training (i.e., aerobic, resistance or both) (Ludyga, 2020). This latter comprehensive review particularly highlighted the benefit on cognition when physical training involved a cognitive component, as opposed to on its own. Another large meta-analysis of randomised controlled trials investigating the most effective physical training modality for enhancing cognition found tai chi (which involves both physical and cognitive processes involved during intricate coordination) and multicomponent physical training (involving cognition) had greater effect sizes than either aerobic or resistance training alone (Northey et al., 2018). More specifically, two systematic reviews (Dong et al., 2020; Lauenroth et al., 2016) and two meta-analyses (Karssemeijer et al., 2017; Zhu et al., 2016) have been conducted to date on combined physical and cognitive training. These have found evidence of cognitive enhancement following training of that specific cognitive skill across all ages (Lauenroth et al., 2016) and global cognitive improvements for healthy older adults (Zhu et al., 2016), older adults with mild cognitive impairment (Dong et al., 2020) and older adults with dementia (Karssemeijer et al., 2017). Thus, whilst still in early stages, the research on combined physical and cognitive training for improving higher order cognitive skills, such as inhibitory control, shows promise.

There is a lack of neuroimaging and electrophysiology research examining the mechanisms of combined physical and cognitive training’s effects on inhibitory control, and results from the small body of work that has been conducted does not provide a clear mechanistic picture. For example, while Adcock et al. (2020) found no changes in brain structure following 16-weeks of exergaming, others have observed exergaming to modulate prefrontal activity and enhancement of inhibitory control (Chen et al., 2017; Eggenberger et al. 2016; Schattin et al., 2016). However, Schattin et al. (2016) found increased prefrontal cortex activity in both the exergaming and the balance training groups, thus this doesn’t clarify the mechanism specific to combined training that may be modulating inhibitory control improvements. Similarly, none of these studies (Adcock et al. e2020; Chen et al., 2017; Eggenberger et al. 2016; Schattin et al., 2016) compared combined training to either physical or cognitive training alone to infer specificity behind the mechanisms of the additive effects. Ji et al. (2019) attempted to address this gap, and found that combined physical and cognitive training, as compared to sedentary reading and just cognitive training, resulted in greater cerebral oxygenation in the left ventrolateral prefrontal cortex during the Stroop task, which was indicative of enhanced inhibitory control. However, this study did not find any differences between the combined training group as compared to physical training alone, which once again doesn’t clarify the additive mechanistic effects of combined training compared to singular physical and cognitive training. Given that there is no other research to our knowledge investigating the effects of combined training compared to singular physical and cognitive training on inhibitory control mechanisms, the unique neuroanatomy of the possible combined mechanism is relatively unknown.

The parameters of physical and cognitive training, such as physical training intensity, the way they have been combined (sequentially or simultaneously) and training durations, are inconsistent across studies, and the outcomes of these variables is unknown. Thus, it is important to give this careful consideration at this early stage of designing and investigating combined training approaches. Additionally, no review to date has examined this approach on any specific cognitive domain. Given the core role that inhibitory control plays in both everyday wellbeing and across numerous mental health and neurological illnesses, analysing whether this training approach can enhance inhibitory control is the next step in uncovering its potential.

### The Current Review

The current meta-analysis aims to investigate the effects of combined physical and cognitive training, relative to control conditions involving no physical or cognitive training, on inhibitory control. Further, the analysis will examine whether outcomes are influenced by training parameters (physical training intensity, number of sessions and whether training was sequential or simultaneous) and whether outcomes differ across participant characteristics (health status and age).

## Method

The study was conducted in accordance with the Preferred Reporting Items for Systematic Review and Meta-Analysis guidelines (PRISMA; Moher, Liberati, Tetzlaff, & Altman, 2009). The prospective study protocol was pre-registered on the National Institute of Health and Research PROSPERO International Prospective Register of Systematic Reviews (Registration number: CRD42019131709).

### Search strategy

An electronic search of online databases including MEDLINE, PsychINFO, Cochrane Central Register of Controlled Trials, Embase and Web of Science (core collection) was conducted to identify relevant literature in November of 2019 and then updated in July 2020 and January 2021. The search strategy for Ovid Cochrane Central Register of Controlled Trials included: ((Combined or Dual-task or Dual task or Multimodal or Multi-modal or Multicomponent or Multi-component) AND exp training/ or exp exercise/ or fitness/ or exp physical activity or (Physical training or Physical activ* or Exercis* or Resistance training or Endurance training or Aerobic training) AND cognition/ or video games/ or (“Cognitive training” or “Brain training”) OR (combined training or exergaming or cognitive-motor)) AND exp behavioural control/ or exp impulsiveness/ or exp inhibition psychology or (Inhibition or Behav* Control or Stop signal or stop-signal or go no-go or go no go or go no-go or go no/go or stroop). See Supplementary Information: Table 1 for search strategies used in each database. Reference lists of included studies were also manually searched for additional articles.

### Eligibility criteria

**Figure 2:**
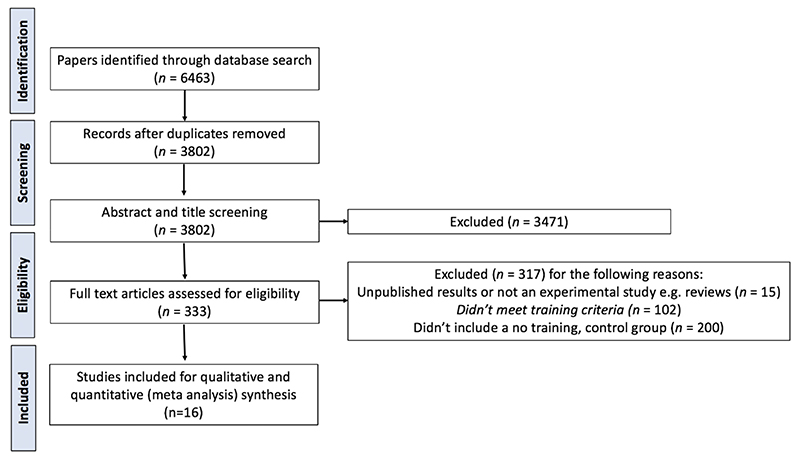
PRISMA flowchart of study identification, screening, assessment of eligibility and inclusion for synthesis

All articles, first based on the title and abstracts and then full text, were screened by two independent reviewers using Covidence (Veritas Health Innovation, 2017). Conflicts were resolved by consultation with a third reviewer and discussed until consensus achieved. Figure 2 displays the process of study identification. This process was repeated in January 2021 and another two recent studies were included, with a final of 16 included studies.

Studies were eligible for inclusion if they: (1) investigated the outcome of combined cognitive and physical training, (2) involved combined training which was simultaneous (cognitive and physical training occurred at the same time) or sequential (physical and cognitive training occurred immediately after each other), (3) investigated the impact of training on one or more measures of inhibitory control, (4) compared outcomes to a comparator (i.e. no physical or cognitive training) condition, (5) involved physical training that comprised repeated engagement in one or more physical activities for at least 20-minutes and (6) involved cognitive training that comprised of repeated engagement in a cognitive task. It’s important to note here that studies which also included an active control group (either physical or cognitive training alone groups) were not excluded, as long as there was still a comparison to an additional passive (no cognitive or physical training) group.

Exclusion criteria included: (1) non-experimental publications, such as reviews, meta-analyses, dissertations, abstracts, non-peer reviewed articles and book chapters, (2) unpublished studies, (3) studies not published in English and (4) studies that did not report extractable data or authors did not provide this when contacted.

### Data extraction

Data extraction included information on study design, participants demographics, recruitment methods, cohort characteristics, training parameters (e.g., physical training intensity, number of sessions and combination method), comparator condition, inhibitory control outcome measure, and data that enabled calculation of study effect sizes (means and standard deviations of pre- and post-training inhibition scores, sample sizes). Where physical training intensity was not reported (*n* = 3 studies), the Compendium of Physical Activities (Ainsworth et al., 2011) guidelines were used to assess MET values of the activities undertaking and low, moderate or vigorous intensities was allocated accordingly.

For studies reporting outcomes for multiple measures of inhibitory control (e.g., the Stroop, No Go/No tasks) the strongest and most comparable measure was included in the meta-analysis. This was determined by selecting the measure which had the most robust psychometric properties, and where these were equivalent, the more commonly used in the literature was included. Only data from the combined physical and cognitive training paradigms were analysed. Where studies reported outcomes from different intervention paradigms (e.g., exercise with non-invasive brain stimulation), these conditions were excluded from the analyses. Outcomes measured immediately post training were analysed, and results obtained at delayed follow up were not included. A single study reported inhibitory control outcomes immediately after both 6- and 12-months of training, and both these time points were included and analysed as separate studies (Suzuki et al., 2012).

### Publication bias

**Figure 3:**
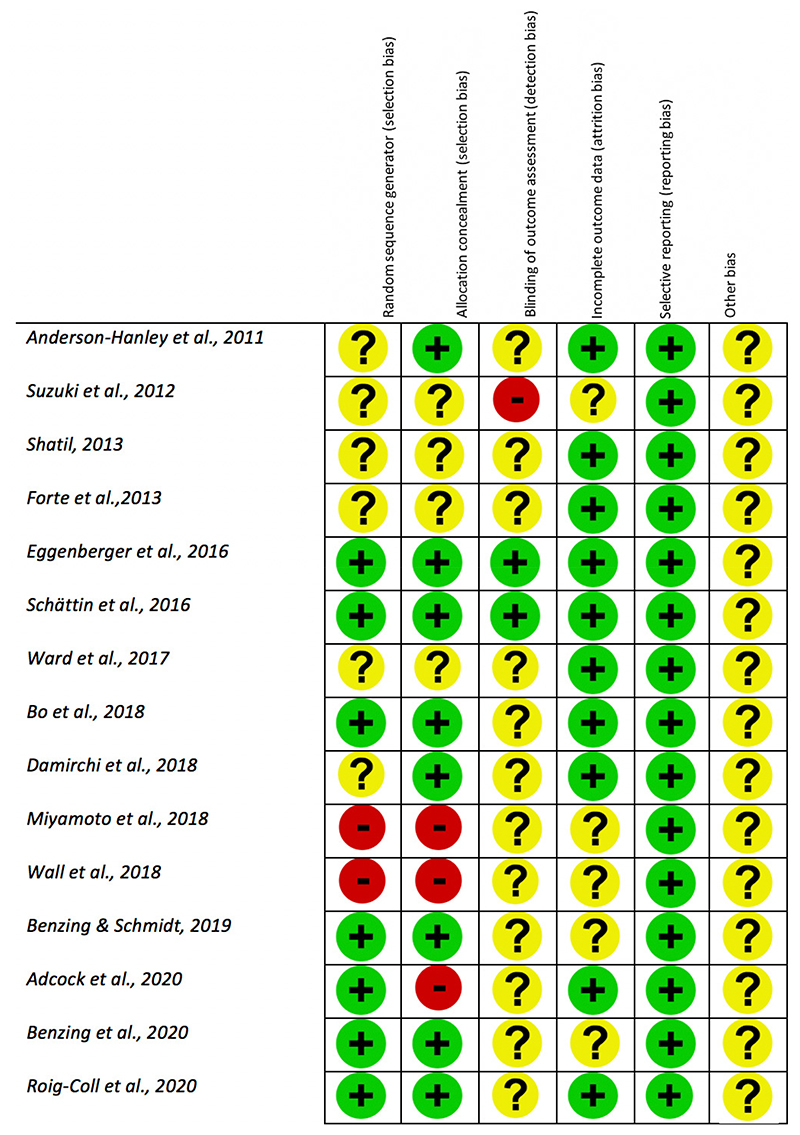
Cochrane’s Quality Assessment. This figure follows the recommendations outlined in the Cochrane Collaboration guidelines (Higgins et al., 2019). A “green plus” indicates a low risk of bias, a “yellow question mark” indicates an unclear risk of bias and a “red minus” indicates some risk. Other risk has been marked as unclear for all studies given it is difficult to make subjective judgments about the involvement of commercial interest across studies, or any other related bias that is not specifically being investigated. Note: this figure requires publishing in colour

Risk of publication bias was evaluated using Egger’s test and funnel plots (Egger, Smith, Schneider, & Minder, 1997); see Supplementary Information: Figure. 1). Egger’s test was non-significant, indicating no significant publication bias, 95% CI [-0.25, 4.37], *t*(2)= 1.91, *p*=0.07. However, the funnel plot revealed that the results of Eggenberger at al. (2016) had a disproportionately large effect size. As such a sensitivity analysis was performed, with the main analysis conducted with and without this study included. Its presence did not change the significance of the results (see Supplementary Information, Figures. 2 and Figure. 3).

### Quality assessment

The risk of biases for each individual study was assessed using the Cochrane Risk of Bias Tool, see Figure 3 (Higgins et al., 2011). Risk was assessed to be ‘low’, ‘high’ or ‘unclear’. Given that all studies included behavioural interventions, particularly physical training, it would not be possible to achieve participant blinding. Therefore, the criteria ‘blinding of participants and personnel (performance bias)’ was omitted from this risk assessment. This resulted in the appearance of higher than usual study quality. One author assessed the risk of biases for all studies. Ambiguities were resolved via discussion with a second author.

### Statistical analysis

#### Calculation and combination of effect sizes

All meta-analyses used random effect models, as implemented in Comprehensive Meta-Analysis Software 3.0 (Borenstein, Hedges, Higgins, & Rothstein, 2013). For inhibitory control outcomes, means, standard deviations and sample sizes for each group were entered to compute the standardised difference in means across groups for each study (*Hedge’s g*). One study (Suzuki et al., 2012) did not report these raw data, therefore the change in means were used to compute the effect size. Hedge’s g values of 0.2, 0.5 and 0.8 served as threshold for small, medium and large effects, respectively (Cohens, 1992). Cochrane’s handbook recommends interpretations as 0-40% not important, 30-60% moderate heterogeneity, 50-90% substantial heterogeneity and 75-100% considerable heterogeneity (Higgins et al., 2019)

#### Main and subgroup analyses

The primary analysis assessed the relationship between training and inhibitory control, and all studies were combined regardless of study characteristics. The secondary, subgroup analyses included investigating whether particular populations characteristics and age groups (children (<10 years old), adolescents (10-18 years old), adults (19-59 years old) and older adults (>60 years old)) responded differently to the training. Additionally, secondary analyses also assessed the training parameters by investigating the effect of training intensities (light, moderate or vigorous), number of sessions (single or multiple sessions) and combination modality (sequential or simultaneous) on inhibitory control.

## Results

### Study Characteristics

The characteristics of studies included in the analyses are shown in Table 1.

### Overall Effects

**Figure 4:**
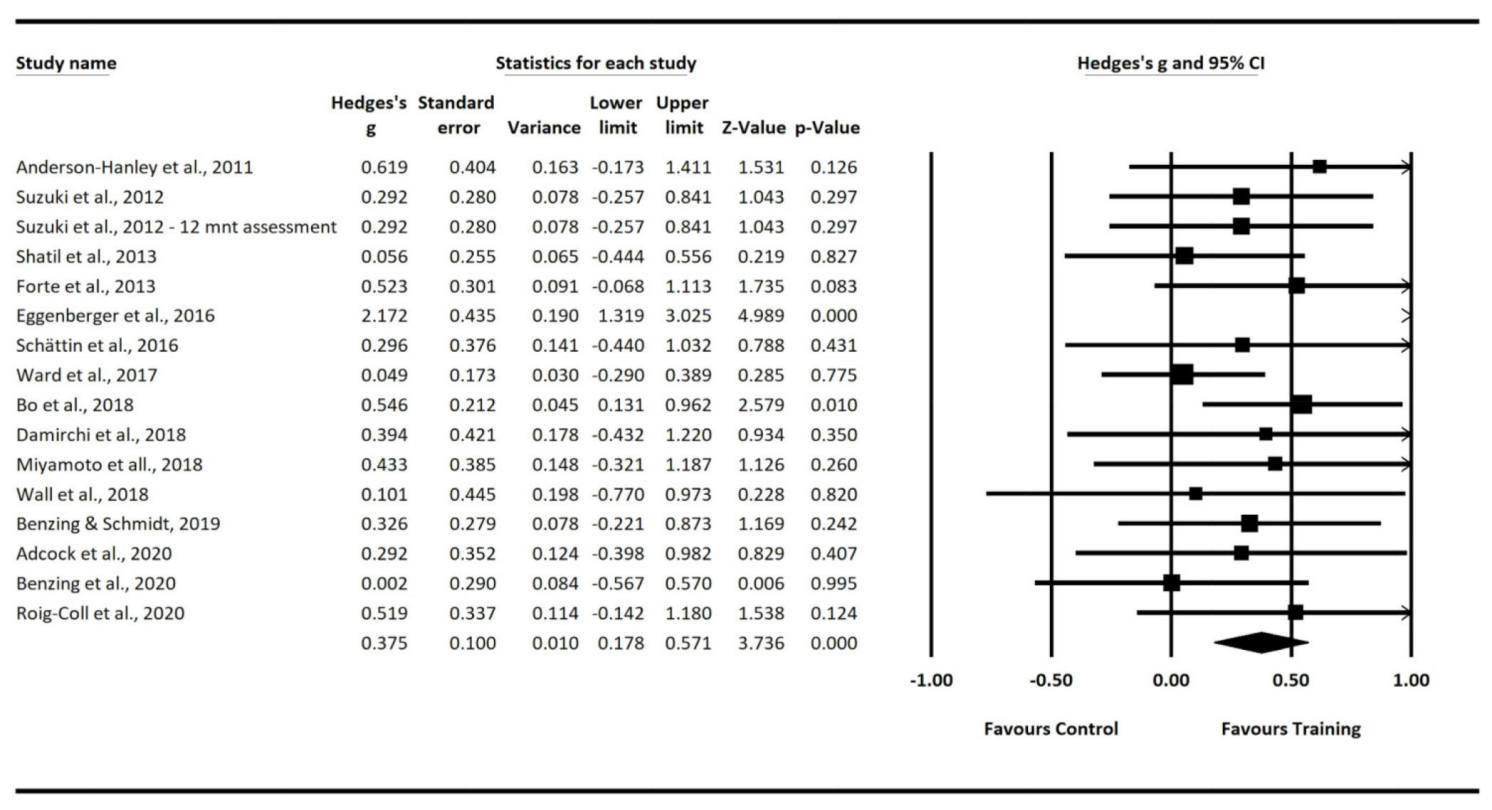
Overall Results.

All the following findings (main effects and sub analyses) are reported relative to control conditions involving no physical or cognitive training (e.g., waitlist control). The summary random effect of combined physical and cognitive training on inhibitory control was small (effect size, *g* = 0.375; 95% CI [0.178,0.561]; *p* < 0.001; Figure 4), with moderate heterogeneity amongst studies (*I*2 = 41.517%) (*n* studies = 16, *n* participants = 832).

### Subgroup Analyses

See supplementary Information: Fig. 5, 6, 7 and 8 for subgroup analyses output and table 2 for summary of subgroup results.

Table 2. Subgroup Analyses on the Outcome of Combined Training across Participant and Training Characteristics

#### Participant Health Status

Subgroup analyses comparing participants with different mental, physical and neurological health statuses revealed that studies investigating individuals with vascular cognitive impairment (*n* studies = 1, *n* sample size = 179) and healthy individuals (*n* studies = 8, *n* sample size = 420) showed significant improvement on inhibitory control from the combined training compared to the control training (*g* = .546; CI [0.131, 0.963]; *p* < 0.001, *g* = .476; CI [0.093, 0.858]; *p* < 0.05, respectively). However, this effect was not significant for studies investigative individuals with ADHD (*n* studies = 1, *n* sample size = 51), ASD (*n* = 1, *n* sample size = 12), cancer survivors (*n* studies = 1, *n* sample size = 69) and those with mild cognitive impairment (*n* studies = 4, *n* sample size = 108), *p* = .242, *p* = .126, *p* = .995 *p* = .091, respectively. There was no heterogeneity across studies investigating individuals with vascular cognitive impairment, ADHD, ASD, cancer survivors and mild cognitive impairment (*I2* = 0%), however studies including healthy individuals demonstrated substantial heterogeneity (*I2* = 68.88%).

#### Participant Age

Subgroup analyses comparing participants of different ages revealed that studies investigating older adults (*n* studies = 10, *n* sample size = 473) showed significant improvement on inhibitory control from the combined training compared to the control training (*g* = .455; CI [0.161, 0.749]; *p* < 0.001). However, this effect was not significant for studies investigating adolescents (*n* studies = 3, *n* sample size = 132) and adults (*n* studies = 3, *n* sample size = 227) (*p* = .150, *p* = .191, respectively). There was no heterogeneity (*I2* = 0%) and low heterogeneity (*I2* = 0.40%) across studies investigating adolescents and adults, however studies including older adults demonstrated moderate heterogeneity (*I2* = 54.73%).

#### Physical Training Intensity

Subgroup analyses comparing different physical training intensities revealed that studies investigating training involving moderate intensity physical training (*n* studies = 12, *n* sample size = 621) showed significant improvement on inhibitory control compared to the control training (*g* = .459; CI [0.220, 0.699]; *p* < 0.001). However, this effect was not significant for studies investigating low (*n* studies = 3, *n* sample size = 79) or vigorous (*n* studies = 1, *n* sample size = 132) physical training intensity (*p* = .717, *p* = .775, respectively). There was no heterogeneity (*I2* = 0%) across studies investigating low and vigorous physical training intensities, however studies including moderate physical training intensity demonstrated moderate heterogeneity (*I2* = 43.74%).

#### Combined Training Number of Sessions

Subgroup analyses comparing different number of training sessions revealed that studies investigating multiple training sessions (*n* studies = 14, *n* sample size = 807) showed significant improvement on inhibitory control compared to the control training (*g* = .364; 95% CI [0.148, 0.581]; *p* < 0.05). However, this effect was not significant for studies investigating single session training paradigms (*n* studies = 2, *n* sample size = 25) (*p* = .061). There was no heterogeneity (*I2* = 0%) across studies investigating single session training, however studies including multiple session training demonstrated moderate heterogeneity (*I2* = 48.12%).

#### Training Combination Modality

Subgroup analyses investigating different combinations of physical and cognitive training revealed that both sequential (immediate succession) (*n* studies = 4, *n* sample size = 365) and simultaneous (occurring at the same time) (*n* studies = 12, *n* sample size = 467) training showed significant improvement on inhibitory control compared to the control training (*g* = .380; 95% CI [0.109, 0.652]; *p* < 0.05; *g* = .389; 95% CI [0.131, 0.648]; *p* < 0.05, respectively). There was no heterogeneity across studies investigating sequential training (*I2* = 0%), however studies including simultaneous training demonstrated moderate heterogeneity (*I2* = 52.373%).

## Discussion

The current meta-analysis is the first synthesis of the rapidly growing body of evidence examining the efficacy of combined physical and cognitive training paradigms to enhance inhibitory control. The primary analysis showed a small positive effect of combined training in increasing inhibitory control, as compared to no training comparator conditions (e.g., waitlist control groups). Secondary analyses, comprising of smaller subgroups, showed a moderate positive effect for older adults, as compared to adolescents and adults; and for populations that were healthy or had vascular cognitive impairment, as compared to those with ADHD and ASD, cancer survivors and those with mild cognitive impairment. Additionally, there was a positive moderate effect when physical training was delivered at moderate intensity, as compared to low or vigorous intensities; a positive small effect when the training involved multiple sessions, as compared to single session paradigms; and a positive small significant effect both when the training was sequentially or simultaneously combined. Collectively the results indicate the importance of considering the individuals most likely to benefit from combined physical and cognitive training (i.e., older adults and healthy individuals) and the most effective training parameters (i.e., moderate intensity physical training, multiple sessions, either sequentially or simultaneously combined cognitive and physical elements) for beneficial outcomes on inhibitory control.

There are currently no effective established behavioural interventions that either specifically target or effectively remediate poor inhibitory control, despite the important role it plays in both health and illness. Whilst the effect was small in magnitude, the current analysis found that combined physical and cognitive training shows capacity to enhance inhibitory control. The potential synergistic mechanisms discussed in the introduction, i.e., physical training inducing a highly neuroplastic environment that may augment the impact of cognitive training on inhibitory control, Figure 1), may underpin this finding (Fabel et al., 2009; Motta-Teixeira et al., 2016; Raichen & Alexander, 2017). In addition to efficacy, a notable benefit of these kinds of behavioural interventions is that they are often highly acceptable to end-users due to a myriad of factors including low side effects, elimination of the need to engage with medical professionals, and high accessibility via at home or community gym engagement (Diepeveen et al., 2013; Devereux-Fitzgerald et al., 2016). Thus, both this initial indication of efficacy in enhancing inhibitory control and the broad appeal and scalability of combined physical and cognitive training paradigms provide a strong rational for ongoing development of this approach.

Sub-group analyses examining whether participant characteristics were associated with training outcomes revealed that the impact of combined training on inhibitory control differed substantially across age. Combined physical and cognitive training had a moderate effect on enhancing inhibitory control in older adults (>60 years old), and no effect in adolescents and adults. There is evidence that inhibitory control declines in older adulthood, compared to early-mid adulthood (Cohn, Dustman, & Bradford, 1984; Nielson, Langenecker & Garavan, 2002), thus this may reflect a greater effect of combined training when impairments in inhibitory control are present at baseline (i.e., training remediating reduced inhibitory control, as opposed to enhancing intact control). However, the observation of a positive medium effect size for healthy individuals (i.e., not likely experiencing impaired inhibitory control) and the lack of apparent efficacy in ADHD and ASD (i.e., developmental disorders characterised by impaired inhibitory control (Geurts, Van Den Bergh & Ruzzano, 2014; Schachar et al., 2000)) is not consistent with the notion that a pre-existing impairment in inhibitory control is likely to show a greater enhancement. These results may reflect the substantially lower power in studies investigating the impact of combined training in ADHD (*n* studies = 1, *n* participants = 51) and ASD (*n* studies = 1, *n* participants = 12), compared to studies investigating older adults (*n* studies = 10, *n* participants 473) and healthy individuals (*n* studies = 8, *n* participants = 420), thus this is best considered as a preliminary effect for ADHD and ASD indications. Additionally, the current analysis was not able to quantify the impact of baseline inhibitory control on training outcomes as this data was unavailable across the included studies. Taken together, it’s unclear whether baseline inhibitory control ability has a true effect on the efficacy of combined training. However, this is important to investigate in future research of combined physical and cognitive training interventions as baseline cognitive ability has been shown to be influential on the outcomes of cognitively focussed interventions (Allott et al., 2020; Guye, De Simoni & Von Basitin, 2017; Lopez et al., 2018). Future research may investigate this by broadening the investigation of combined physical and cognitive training to indications that are less powered across this review and/or analysing the influence of baseline cognitive capacity on outcomes of training.

In addition to age, differing outcomes were observed across studies of different health or illness indications. Studies investigating the impact of combined training in healthy individuals and those with vascular cognitive impairment showed moderate effects in improving inhibitory control, whereas studies focussing on ADHD, ASD, cancer survivors and mild cognitive impairment did not show an effect of training. The positive effect observed for vascular cognitive impairment (*n* studies = 1, *n* participants = 179) and the nonsignificant effects on cancer survivors (*n* studies = 1, *n* participants = 69) and those with mild cognitive impairment (*n* = 4, *n* participants = 108) should be interpreted with some caution as they are based on a minimal, albeit well powered, investigations. The positive, moderate effect size and largest body of evidence in this review, thus most powered, is that healthy individuals (*n* studies = 8, *n* participants = 420) benefit from the combined training. This has promising implications for the capacity of combined training to enhance general wellbeing as greater inhibitory control is associated with strong life satisfaction (Lee & Chao, 2012), mindfulness (Oberle, Schonert-Reichl, Lawlor & Thomson, 2012), and emotional regulation (Hudson & Jacques, 2014). Thus, whilst combined physical and cognitive training may be particularly effective when inhibition is impaired (i.e., in older adults due to ageing), the current results indicate that there is also capacity for enhancement of healthy inhibitory control systems.

The intensity of physical training that was delivered alongside cognitive training in the combined paradigms was highly influential. Training protocols employing moderate intensity exercise had a moderate positive effect on inhibitory control, whereas no significant effects were observed when low or vigorous intensity was used. The finding that, when combined with cognitive training, moderate intensity physical exercise is the most associated with inhibitory control improvements is inconsistent with recent evidence indicating that vigorous intensity exercise is most effective for acute pose-exercise cognitive enhancement (Coetsee & Terblanche, 2017; Coxon et al., 2018; Saucedo et al., 2015). However, the fatigue from vigorous physical training may lead to difficulty reserving energy for engagement in cognitive training, thus leading to a potential inverse effect of cognitive decline due to potential cognitive overload. Thus, moderate intensity physical training appears to be an optimal middle ground for synergistic neurocognitive benefits from combined training because (1) as compared to low intensity physical training, it may provide the sufficient intensity to induce the broad neuroplastic effects necessary for cognitive enhancements, and (2) as compared to vigorous intensity, it may allow energy to be reserved for effective engagement in across both physical and cognitive training. The potent influence of physical training intensity on cognitive outcomes highlights the importance of training parameters, and of prescribing this accurately in future studies. However, the clear heterogenicity across intensities prescribed and outcomes reported in current physical training studies highlights the lack of specific prescription of intensity. Moving forward, future combined physical and cognitive training studies targeting cognitive skills, such as inhibition control, will benefit from using methods of physical training intensity personalisation (e.g., personalised fitness testing such as VO2 max assessments, heart rate monitors, chest straps) to ensure that physical training is moderately intense across individuals.

Positive impacts of combined training on inhibitory control were observed after multiple sessions of training (range = 3 sessions p/w for 8 weeks – 2 sessions p/w for 12 months), but not after single sessions. While intuitive, this observation has methodological implications for studies employing single session experimental designs. Single session proof-of-concept studies are often used when exploring the merit of new intervention approaches or testing the implications of parameter modifications (Armitage et al., 2020; Radley-Crabb et al., 2012; Turton et al., 2018). That the current meta-analysis found no evidence of effects on inhibitory control following a single session of combined physical and cognitive training indicates that either more sessions are needed for modulation to occur, or far more sensitive outcome measures are required to detect subtle effects. The majority of studies in the current analysis used the Stroop as the primary measure of inhibitory control, specifically the interference score. While commonly used clinically and in research, the Stroop is relatively insensitive to subtle cognitive change and there may be value in using more sensitive neurocognitive measure (e.g., evoked potentials, eye tracking tasks) of inhibitory control in future single session research designs. Finally, that effects on inhibitory control were equivalent when the cognitive and physical training elements were delivered simultaneously or sequentially is a useful observation for future intervention designers as it provides flexibility in the ways that cognitive and physical training elements are combined.

While the focus of the current meta-analysis is topical and the analysis was conducted with methodological rigor, the findings should be considered in light of a number of limitations. Firstly, there was significant heterogeneity in methodologies across the studies included. This included differences in training paradigms, clinical / health indications, and study quality. Whilst the review analysed the impacts of this variability on training outcomes, continued investigation into the parameters and populations most associated with the efficacy of combined physical and cognitive training for inhibitory control is important at this early stage of development. Another important consideration is the omission of participant blinding domains in the risk of bias tool, and the consequential inflation of methodological quality. Whilst it is often not possible to blind participants to the intervention in behavioural trials, possible participant bias is important to consider when drawing implications, particularly across studies where participants weren’t blinded to the outcomes assessed. Additionally, as noted above, the observation of greater training efficacy in those domains that had the largest bodies of evidence (i.e., older adults, healthy individuals, moderate intensity physical training) highlights the need for further evidence collected with larger sample sizes and replication of this meta-analysis at that time. As the number of investigations into combined training for inhibitory control is growing rapidly, this may not be far off. The current review provides the first step in this process and is timely given the substantial interest and promise in this novel intervention approach.

An important consideration within this topic of research is the current lack of understanding regarding the mechanisms of combined physical and cognitive training on inhibitory control networks. There have been few neurophysiological studies investigating combined physical and cognitive training, and even fewer focused specifically on inhibitory control networks. This meta-analysis was a first step in evaluating the effects of combined physical and cognitive training on improving inhibitory control, and as such did not aim to elucidate the neuroanatomical underpinnings of this approach. Going forward, however, this will be an important area of basic research and necessary to progress the field and keep pace with the rapid proliferation of efficacy investigations.

A final important consideration is the nature of the comparator condition employed by studies included in the analysis. While uncontrolled studies were excluded from the analysis in an attempt to maximise veracity, the comparator of those that were included often invariably involved an absence of physical or cognitive training (e.g., waitlist control) as opposed to comparison with cognitive training-alone or physical training-alone. Although this was consistent with The National Institutes of Health Office of Behavioral and Social Science Research expert panel’s recommendations that waitlist, or no treatment comparators are sufficient candidates when the purpose of the behavioural trial is to examine whether there is any initial promise of efficacy (i.e., “whether it works at all” (pg. 79), this is was a notable limitation of the current literature. Only 5 of the 15 studies compared combined physical and cognitive training to cognitive and physical training alone, with the remaining employing waitlist or no training comparator conditions. The lack of widespread comparison to singular physical or cognitive training paradigms makes it difficult to infer whether effects are due to the *combination* of physical and cognitive training, as opposed to either activity alone. Thus, further work is required to quantify and parcellate the relative contributions of cognitive training, physical training, and dual training on inhibitory control.

In summary, the current meta-analysis found small significant positive effects of combined physical and cognitive training on improving inhibitory control, as compared to control conditions involving no physical or cognitive training. Greater efficacy was observed when training was delivered to older adults and healthy individuals and when physical training was of moderate intensity. The sequence in which cognitive and physical training was administered did not impact outcomes, and single training sessions did not reliably induce observable improvements on inhibitory control. The analysis sheds light on the importance of both training parameters and individual differences in relation to efficacy. Overall, the data supports the value of ongoing development of this novel and neurobiological plausible approach to cognitive enhancement. With further development, combined physical and cognitive training has potential to cut across mental, physical and neurological symptoms associated with inhibitory control impairments.

## Supplementary Material

Supplementary Information

## Figures and Tables

**Table 1:** Demographics and Study Characteristics

Study	Sample Size	Age	Gender	Cohort details	Inhibition measure	Type of combined training	Session Dose	Combination modality	Physical training modality	Physical training intensity: intensity personal isation method	Cognitive training modality
Anderson-Hanley et al., 2011	12	Adolescent	f = 4, m = 8	Autism Spectrum Disorder	Stroop	Dance Revolution	Single Session	Simultaneous	Aerobic: dance	Low: not reported	Gaming: imitating dance moves
Suzuki et al., 2012	50	Older adult	f = 23, m = 37	Mild Cognitive Impairment	Stroop	Traditional exercises + cognitive training	Multiple Session	Simultaneous	Strength + balance: circuit training, stair stepping and walking	Moderate: 60% of max heart rate	Cognitive tasks (e.g. creating a poem whilst exercising)
Suzuki et al., 2012	50	Older adult	f = 23, m = 37	Mild Cognitive Impairment	Stroop	Traditional exercises + cognitive training	Multiple Session	Simultaneous	Strength + balance: circuit training including stair stepping, endurance walking, and walking.	Moderate: 60% of max heart rate	Varied of cognitive tasks (e.g. creating a poem whilst exercising)
Shatil, E., 2013	60	Older adult	f = 39, m =11	Healthy	Stroop	Traditional exercises + cognitive training	Multiple Session	Sequential	Aerobic + strength + flexibility: warm up, full body movements and a cooldown.	Low: Not reported	Cognitive training: 21 tasks in the CogniFit personalized regiment.
*Forte et al., 2013*	42	Older adult	f = 26, m = 16	Healthy	The Runs	Traditional exercises + cognitive training	Multiple Session	Simultaneous	Strength + stretching + balance: walking progressively at faster speeds, skipping and jogging, while adding the use of the arms	Moderate : 15-17 on Borg scale of perceived exertion	Cognitive tasks: specific cognitive challenges to engage executive function, and to specifically stimulate the inhibition of habitual responses and cognitive flexibility
Eggenberger etal., 2016	33	Older adult	f = 21, m = 12	Healthy	Stroop	Dance game	Multiple Session	Simultaneous	Dance	Moderate : training adapted to each participant’s abilities to achieve a moderate-vigorous intensity.	Gaming: attention demanding cognitive task.
*Schättin et al., 2016*	27	Older adult	f = 12, m = 15	Healthy	Go-No/Go	Exergaming	Multiple Session	Simultaneous	Dance	Moderate: intensity was based on studies illustrating positive training effects in older adults and on current recomme ndations for achieving physical fitness and fall prevention in elderly	Gaming: participants performed specific whole body movements driven by a video game presented on a frontal scree
Ward etal., 2017	132	Adult	f = 61, m = 71	Healthy	Stroop	Gaming	Multiple Session	Simultaneous	Aerobic + strength: dynamic stretchiest, walk/run, high intensity cardiovascular and resistance training.	Vigorous: % HR monitored to ensure high intensity	Gaming: six games all based on psychological tasks of executive function and working memory
*Bo et al., 2018*	179	Older adult	f = 79, m = 100	Vascular Cognitive Impairment	Stroop	Traditional aerobic exercise + cognitive training	Multiple Session	Sequential	Aerobic + endurance: jogging/cycling, strength and stretching	Moderate: ratings of 13-15 on Borg scale of perceived exertion	Cognitive training: total set of 12 exercises on iPads including tasks of execution function, attention and speed.
*Damirchi et al., 2018*	44	Older adult	f = 44, m = 0	Mild Cognitive Impairment	Stroop	Traditional aerobic exercise + cognitive training	Multiple Session	Sequential	Aerobic + strength: complex body motion exercises and light cardio.	Moderate 55-75% of max hr	Cognitive training: four games. (1) Photo flaw: engaged visual attention, visual working memory, and speed of processing. (2) Headline Clues: focused on anecdotal knowledge, engaging verbal memory, and reasoning. (3) Sokoban: exercised strategic planning and both spatial executive processing and visual spatial skills. (4) Keep It In Mind: for assessing working memory.
*Miyamo to et al., 2018*	13	Adult	f = 0, m = 13	Healthy	Stroop	Cycling + cognitive training	Single Session	Simultaneous	Cycling	Moderate : 60% VO2peak	Cognitive training: tasks of executive functions and memory
*Wall etal., 2018*	14	Older adult	f = 6, m = 8	Mild Cognitive Impairment	Stroop	Traditional exercises + gaming	Multiple Session	Simultaneous	Cycling	Low: intensity measure ment not reported	Gaming: executive functions by simulating the naturalistic task of traveling along a roadway to complete a given list of errands and then returning.
*Benzing & Schmidt, 2019*	51	Adolesce nt	f = 9, m = 42	Attention Deficit Hyperactivity Disorder	Simon Task	Xbox Kinect	Multiple Session	Simultaneous	Strength + endurance: interaction with the console through body movement	Moderate: Adaptive difficulty – no measure of exercise intensity	Gaming: demands cognitive functions such as inhibition, switching, updating, attention, and speed of action.
*Adcock et al., 2020*	31	Older adult	f = 16, m = 15	Healthy	Stroop	Exergaming	Multiple Session	Simultaneous	Tai Chi	Moderate : progressive difficulty – current difficulty level should always provide an optimal challenge avoiding under- or overload	Gaming: by stepping forward, backward, and to the right or left side, the cognitive games are played and controlled
*Benzing et al., 2020*	69	Adolescent	f = 21, m = 48	Cancer Survivors	Stroop	Exergaming	Multiple	Simultaneous	Strength, coordination and endurance	Moderate	Gaming: activities requiring executive functions
*Roig-Coll et al., 2020*	82	Adults	f = 51, m = 31	Healthy	Stroop	Traditional aerobic exercise + cognitive training	Multiple session	Sequential	Brisk walking	Moderate: 12-14 on Borg scale of perceived exertion	Computerized home based games using the Gutmann Neuropers onal Trainer

**Table 2:** Subgroup Analyses on the Outcome of Combined Training across Participant and Training Characteristics

Subgroup Population Characteristics	*n* (studies)	*n* (participants)	*p*	Hedge’s *g*	Heterogeneity (%)
Participant Health Status					
Healthy Individuals (all ages)	8	420	<0.001	.546	68.88%
Vascular Cognitive Impairment	1	179	<0.001	.476	N/A
ADHD	1	51	.242	*ns*	N/A
ASD	1	12	.126	*ns*	N/A
Cancer Survivors	1	69	.995	*ns*	N/A
Mild Cognitive Impairment	4	108	.091	*ns*	0%
Participant Age					
Older Adults	10	473	<.001	.455	54.73%
Adults	3	227	.191	*ns*	.4%
Adolescents	3	132	.150	*ns*	0%
Training Characteristics					
Physical Training Intensity					
Vigorous Intensity	1	132	.775	*ns*	N/A
Moderate Intensity	12	621	<.001	.459	43.74%
Low intensity	3	79	.717	*ns*	0%
Number of sessions					
Multiple	14	807	<.05	.364	48.12%
Single	2	25	.061	*ns*	0%
Training Combination Modality					
Simultaneous	12	467	.05	.389	52.37%
Sequential	4	365	<.05	.380	0%

## Data Availability

Available upon request
