# Supplementary material for: The Effects of Combined Physical and Cognitive Training on Inhibitory Control: A Systematic Review and Meta-Analysis
Source: Neurosci Biobehav Rev. Author manuscript; Available in PMC 2021 Sep 1. (PMC7611490; doi:10.1016/j.neubiorev.2021.07.008)
Supplement: Supplementary Information [file EMS132032-supplement-Supplementary_Information.docx]

**Appendix**

**Supplementary Information**

Table 1. Search strategy for each database

| ***Database Field Guide Ovid MEDLINE(R) and Epub Ahead of Print, In-Process & Other Non-Indexed Citations, Daily and Versions*** | | |
| --- | --- | --- |
| **Concept** | **Additional keywords** | **Subject headings** |
| 1- Combined | Combined  Dual-task  Dual task  Multimodal  Multi-modal  Multicomponent  Multi-component | Combined modality therapy/ |
| 2 - Physical training | Physical training  Physical activit*  Exercis*  Resistance training  Endurance training  Aerobic training | exp Exercise/  Physical exertion/  Physical fitness/ |
| 3- Cognitive training | Cognitive training  Brain training | Cognition/  Video games/ |
| 4 – Combined training | Combined training  Exergaming  Cognitive-motor | exp Exercise therapy/ |
| 5- Inhibition | Inhibition  Behav* Control  Stop signal  Stop-signal  Go-no-go  Go no-go  Go no/go  Go no no  Stroop | exp Inhibition (Psychology)/  exp Behavior control/  exp Impulsive behavior/ |
| **Summarised search:**  ((1 AND 2 AND 3) OR 4) AND 5  **((Set 1**  Combined modality therapy/ or (Combined or Dual-task or Dual task or Multimodal or Multi-modal or Multicomponent or Multi-component).mp. [mp=title, abstract, original title, name of substance word, subject heading word, floating sub-heading word, keyword heading word, organism supplementary concept word, protocol supplementary concept word, rare disease supplementary concept word, unique identifier, synonyms]  *AND*  **Set 2**  exp exercise/ or physical exertion/ or physical fitness/ or (Physical training or Physical activ* or Exercis* or Resistance training or Endurance training or Aerobic training).mp. [mp=title, abstract, original title, name of substance word, subject heading word, floating sub-heading word, keyword heading word, organism supplementary concept word, protocol supplementary concept word, rare disease supplementary concept word, unique identifier, synonyms]  *AND*  **Set 3)**  cognition/ or video games/ or ("Cognitive training" or "Brain training").mp. [mp=title, abstract, original title, name of substance word, subject heading word, floating sub-heading word, keyword heading word, organism supplementary concept word, protocol supplementary concept word, rare disease supplementary concept word, unique identifier, synonyms]  *OR*  **Set 4)**  Exp exercise therapy/ or (combined training or exergaming or cognitive-motor).mp. [mp=title, abstract, original title, name of substance word, subject heading word, floating sub-heading word, keyword heading word, organism supplementary concept word, protocol supplementary concept word, rare disease supplementary concept word, unique identifier, synonyms]  *AND*  **Set 5)**  exp inhibition (Psychology)/ or exp behavior control/ or exp impulsive behavior/ or (Inhibition or Behav* Control or Stop signal or stop-signal Go no-go or go no/go go no go or stroop).mp. [mp=title, abstract, original title, name of substance word, subject heading word, floating sub-heading word, keyword heading word, organism supplementary concept word, protocol supplementary concept word, rare disease supplementary concept word, unique identifier, synonyms] | | |
| ***Ovid: PsychINFO*** | | |
| **Concept** | **Additional keywords** | **Subject headings** |
| 1- Combined | Combined  Dual-task  Dual task  Multimodal  Multi-modal  Multicomponent  Multi-component | Multimodal treatment approach/ |
| 2 - Physical training | Physical training  Physical activit*  Exercis*  Resistance training  Endurance training  Aerobic training | exp Exercise/  exp Physical activity/  Training/  exp Physical fitness/  exp Aerobic exercise/ |
| 3- Cognitive training | Cognitive training  Brain training  Video games | Cognition/  Computer games/  exp Brain training/ |
| 4 – Combined training | Combined training  Exergaming  Cognitive-motor | exp physical therapy/  exp perceptual motor processes/ |
| 5- Inhibition | Inhibition  Behav* Control  Stop signal  Stop-signal  Go-no-go  Go no-go  Go no/go  Go no go  Stroop | exp Behavioural inihibition/  exp Behavioural inhibition system/  exp Inhibition (Personality)/  exp Latent inhibition/  exp Response inhibition/  exp Obsessive Compulsive Disorder/  exp Impulsiveness/ |
| **Summarised search:**  ((1 and 2 and 3) or 4) and 5  **1** = Multimodal treatment approach/ or (Combined or Dual-task or Dual task or Multimodal or Multi-modal or Multicomponent or Multi-component).mp. [mp=title, abstract, heading word, table of contents, key concepts, original title, tests & measures, mesh]  **2** = exp exercise/ or exp physical activity/ or training/ or exp physical fitness/ or exp aerobic exercise/ or (Physical training or Physical activ* or Exercis* or Resistance training or Endurance training or Aerobic training).mp. [mp=title, abstract, heading word, table of contents, key concepts, original title, tests & measures, mesh]  **3** = cognition/ or computer games/ or exp brain training/ or ("Cognitive training" or "Brain training" or video games).mp. [mp=title, abstract, heading word, table of contents, key concepts, original title, tests & measures, mesh]  **4** = exp physical therapy/ or exp perceptual motor processes/ or (combined training or exergaming or cognitive-motor).mp. [mp=title, abstract, heading word, table of contents, key concepts, original title, tests & measures, mesh]  **5** = exp behavioural inhibition/ or exp behavioural inhibition system/ or exp inhibition Personality/ or exp latent inhibition/ or exp response inhibition/ or exp obsessive compulsive disorder/ or exp impulsiveness/ or (Inhibition or Behav* Control or Stop signal or stop-signal or Go no-go or go no go or go no-go or go no/go or stroop).mp. [mp=title, abstract, heading word, table of contents, key concepts, original title, tests & measures, mesh] | | |
| ***Ovid: Cochrane Central Register of Controlled Trials*** | | |
| **Concept** | **Additional keywords** | **Subject headings** |
| 1- Combined | Combined  Dual-task  Dual task  Multimodal  Multi-modal  Multicomponent  Multi-component | Combined modality therapy/ |
| 2 - Physical training | Physical training  Physical activit*  Exercis*  Resistance training  Endurance training  Aerobic training | exp Exercise/  Physical exertion/  Physical fitness/ |
| 3- Cognitive training | Cognitive training  Brain training | Cognition/  Video games/ |
| 4 – Combined training | Combined training  Exergaming  Cognitive-motor | exp Exercise therapy/ |
| 5- Inhibition | Inhibition  Behav* Control  Stop signal  Stop-signal  Go-no-go  Go no-go  Go no/go  Go no no  Stroop | exp Inhibition (Psychology)/  exp Behavior control/  exp Impulsive behavior/ |
| **Summarised search:**  ((1 and 2 and 3) or 4) and 5  **1** = Combined modality therapy/ or (Combined or Dual-task or Dual task or Multimodal or Multi-modal or Multicomponent or Multi-component)  **2** = exp exercise/ or exp physical exersion/ or physical fitness/ or (Physical training or Physical activ* or Exercis* or Resistance training or Endurance training or Aerobic training)  **3** = cognition/ or video games/ or ("Cognitive training" or "Brain training")  **4** = exp exercise therapy/ or (combined training or exergaming or cognitive-motor)  **5** = exp behavioural control/ or exp impulsive behavior/ or exp inhibition psychology or (Inhibition or Behav* Control or Stop signal or stop-signal or go no-go or go no go or go no-go or go no/go or stroop) | | |
| ***Ovid: Embase*** | | |
| **Concept** | **Additional keywords** | **Subject headings** |
| 1- Combined | Combined  Dual-task  Dual task  Multimodal  Multi-modal  Multicomponent  Multi-component |  |
| 2 - Physical training | Physical training  Physical activit*  Exercis*  Resistance training  Endurance training  Aerobic training | exp training/  exp exercise/  fitness/  exp physical activity |
| 3- Cognitive training | Cognitive training  Brain training | Exp Cognition/  Exp video game/ |
| 4 – Combined training | Combined training  Exergaming  Cognitive-motor |  |
| 5- Inhibition | Inhibition  Behav* Control  Stop signal  Stop-signal  Go-no-go  Go no-go  Go no/go  Go no no  Stroop | exp Inhibition (Psychology)/  exp Behavior control/  exp Impulsiveness/ |
| **Summarised search:**  ((1 AND 2 AND 3) OR 4) AND 5  **1**= (Combined or Dual-task or Dual task or Multimodal or Multi-modal or Multicomponent or Multi-component)  **2** = exp training/ or exp exercise/ or fitness/ or exp physical activity or (Physical training or Physical activ* or Exercis* or Resistance training or Endurance training or Aerobic training)  **3** = cognition/ or video games/ or ("Cognitive training" or "Brain training")  **4**= (combined training or exergaming or cognitive-motor)  **5** = exp behavioural control/ or exp impulsiveness/ or exp inhibition psychology or (Inhibition or Behav* Control or Stop signal or stop-signal or go no-go or go no go or go no-go or go no/go or stroop) | | |
| ***Web of Science – core collection***  **Summarised search (search format differs to other databases)**  (Combined modality therapy/ or (Combined or Dual-task or Dual task or Multimodal or Multi-modal or Multicomponent or Multi-component).) *AND* **TOPIC:** (exp exercise/ or physical exertion/ or physical fitness/ or (Physical training or Physical activ* or Exercis* or Resistance training or Endurance training or Aerobic training).) *AND***TOPIC:** (cognition/ or video games/ or ("Cognitive training" or "Brain training")) *OR* **TOPIC:** (Exp exercise therapy/ or (combined training or exergaming or cognitive-motor).) *AND* **TOPIC:** (exp inhibition (Psychology)/ or exp behavior control/ or exp impulsive behavior/ or (Inhibition or Behav* Control or Stop signal or stop-signal Go no-go or go no/go go no go or stroop).) | | |

*Figure 1*. Egger’s test and Funnel Plot to assess publication bias

*Figure 2*. Main Results

*Figure 3*. Main Results without Eggenberger et al. included

*Figure 4*. Sub Group Analysis: Population Group

*Figure 5*. Sub Group Analysis: Age

*Figure 6*. Sub Group Analysis: Physical Training Intensity

*Figure 7*. Sub Group Analysis: Combined Training Session Dose

*Figure 8.* Sub Group Analysis: Training Combination Modality

**References**

Abrantes, A. M., Brown, R. A., Strong, D. R., McLaughlin, N., Garnaat, S. L., Mancebo, M., ... & Greenberg, B. D. (2017). A pilot randomized controlled trial of aerobic exercise as an adjunct to OCD treatment. *General hospital psychiatry*, *49*, 51-55. https://doi.org/10.1016/j.genhosppsych.2017.06.010

Ainsworth, B. E., Haskell, W. L., Herrmann, S. D., Meckes, N., Bassett Jr, D. R., Tudor-Locke, C., ... & Leon, A. S. (2011). Compendium of Physical Activities: a second update of codes and MET values. *Medicine & science in sports & exercise*, *43*(8), 1575-1581.

Allott, K., van-der-EL, K., Bryce, S., Parrish, E. M., McGurk, S. R., Hetrick, S., ... & Velligan, D. (2020). Compensatory Interventions for Cognitive Impairments in Psychosis: A Systematic Review and Meta-Analysis. *Schizophrenia bulletin*, *46*(4), 869-883.  https://doi-org.ezproxy.lib.monash.edu.au/10.1093/schbul/sbz134

Anderson-Hanley, C., Arciero, P. J., Brickman, A. M., Nimon, J. P., Okuma, N., Westen, S. C., ... & Zimmerman, E. A. (2012). Exergaming and older adult cognition: a cluster randomized clinical trial. *American journal of preventive medicine*, *42*(2), 109-119. https://doi.org/10.1016/j.amepre.2011.10.016

Armitage, C. J., Walsh, T., Mooney, J., Tierney, S., & Callery, P. (2020). Proof of concept trial for a new theory-based intervention to promote child and adult behavior change. *Journal of behavioral medicine*, *43*(1), 80-87. https://doi-org.ezproxy.lib.monash.edu.au/10.1007/s10865-019-00061-0

Aron, A. R. (2007). The neural basis of inhibition in cognitive control. *Neuroscientist, 13*(3), 214-228. doi:10.1177/1073858407299288

Aron, A. R., Fletcher, P. C., Bullmore, E. T., Sahakian, B. J., & Robbins, T. W. (2003). Stop-signal inhibition disrupted by damage to right inferior frontal gyrus in humans. *Nature Neuroscience, 6*(2), 115-116. doi:10.1038/nn1003

Aron, A. R., Robbins, T. W., & Poldrack, R. A. (2014). Inhibition and the right inferior frontal cortex: one decade on. *Trends Cogn Sci, 18*(4), 177-185. doi:10.1016/j.tics.2013.12.003

Aron, A. R., Robbins, T. W., & Poldrack, R. A. (2014). Inhibition and the right inferior frontal cortex: one decade on. *Trends in cognitive sciences*, *18*(4), 177-185. https://doi.org/10.1016/j.tics.2013.12.003

Barcelos, N., Shah, N., Cohen, K., Hogan, M. J., Mulkerrin, E., Arciero, P. J., . . . Anderson-Hanley, C. (2015). Aerobic and Cognitive Exercise (ACE) Pilot Study for Older Adults: Executive Function Improves with Cognitive Challenge While Exergaming. *J Int Neuropsychol Soc, 21*(10), 768-779. doi:10.1017/S1355617715001083

Bari, A., & Robbins, T. W. (2013). Inhibition and impulsivity: Behavioral and neural basis of response control. *Progress in Neurobiology, 108*, 44-79. doi:10.1016/j.pneurobio.2013.06.005

Barkley, R. A. (1997). Behavioral inhibition, sustained attention, and executive functions: constructing a unifying theory of ADHD. *Psychological bulletin*, *121*(1), 65. https://doi-org.ezproxy.lib.monash.edu.au/10.1037/0033-2909.121.1.65

Berlin, G. S., & Lee, H. J. (2018). Response inhibition and error-monitoring processes in individuals with obsessive-compulsive disorder. *Journal of Obsessive-Compulsive and Related Disorders, 16*, 21-27. doi:10.1016/j.jocrd.2017.11.001

Bird, C. M., & Burgess, N. (2008). The hippocampus and memory: insights from spatial processing. *Nature Reviews Neuroscience*, *9*(3), 182-194. https://doi-org.ezproxy.lib.monash.edu.au/10.1038/nrn2335

Boraxbekk, C. J., Salami, A., Wåhlin, A., & Nyberg, L. (2016). Physical activity over a decade modifies age-related decline in perfusion, gray matter volume, and functional connectivity of the posterior default-mode network—A multimodal approach. *Neuroimage*, *131*, 133-141. https://doi.org/10.1016/j.neuroimage.2015.12.010

Brown, J., Cooper‐Kuhn, C. M., Kempermann, G., Van Praag, H., Winkler, J., Gage, F. H., & Kuhn, H. G. (2003). Enriched environment and physical activity stimulate hippocampal but not olfactory bulb neurogenesis. *European journal of Neuroscience,* 17(10), 2042-2046. https://doi-org.ezproxy.lib.monash.edu.au/10.1046/j.1460-9568.2003.02647.x

Carro, E., Trejo, J. L., Busiguina, S., & Torres-Aleman, I. (2001). Circulating insulin-like growth factor I mediates the protective effects of physical exercise against brain insults of different etiology and anatomy. *Journal of Neuroscience*, *21*(15), 5678-5684. https://doi.org/10.1523/JNEUROSCI.21-15-05678.2001

Castro-Alamancos, M. A., & Connors, B. W. (1996). Short-term synaptic enhancement and long-term potentiation in neocortex. *Proceedings of the National Academy of Sciences*, *93*(3), 1335-1339.  https://doi-org.ezproxy.lib.monash.edu.au/10.1073/pnas.93.3.1335

Castro-Alamancos, M. A., Donoghue, J. P., & Connors, B. W. (1995). Different forms of synaptic plasticity in somatosensory and motor areas of the neocortex. *Journal of Neuroscience*, *15*(7), 5324-5333. https://doi.org/10.1523/JNEUROSCI.15-07-05324.1995

Chaddock-Heyman, L., Erickson, K. I., Voss, M., Knecht, A., Pontifex, M. B., Castelli, D., ... & Kramer, A. (2013). The effects of physical activity on functional MRI activation associated with cognitive control in children: a randomized controlled intervention. *Frontiers in human neuroscience*, *7*, 72. https://doi.org/10.3389/fnhum.2013.00072

Chamberlain, S. R., Blackwell, A. D., Fineberg, N. A., Robbins, T. W., & Sahakian, B. J. (2005). The neuropsychology of obsessive compulsive disorder: the importance of failures in cognitive and behavioural inhibition as candidate endophenotypic markers. *Neurosci Biobehav Rev, 29*(3), 399-419. doi:10.1016/j.neubiorev.2004.11.006

Chang, Y. K., Labban, J. D., Gapin, J. I., & Etnier, J. L. (2012). The effects of acute exercise on cognitive performance: a meta-analysis. *Brain research*, *1453*, 87-101. https://doi.org/10.1016/j.brainres.2012.02.068

Chapman, S. B., Aslan, S., Spence, J. S., DeFina, L. F., Keebler, M. W., Didehbani, N., & Lu, H. (2013). Shorter term aerobic exercise improves brain, cognition, and cardiovascular fitness in aging. *Frontiers in aging neuroscience*, *5*, 75. https://doi.org/10.3389/fnagi.2013.00075

Chekroud, S. R., Gueorguieva, R., Zheutlin, A. B., Paulus, M., Krumholz, H. M., Krystal, J. H., & Chekroud, A. M. (2018). Association between physical exercise and mental health in 1· 2 million individuals in the USA between 2011 and 2015: a cross-sectional study. *The Lancet Psychiatry*, *5*(9), 739-746. https://doi.org/10.1016/S2215-0366(18)30227-X

Chen, T., Yue, G. H., Tian, Y., & Jiang, C. (2017). Baduanjin mind-body intervention improves the executive control function. *Frontiers in Psychology,* 7, 2015.  https://doi.org/10.3389/fpsyg.2016.02015

Coetsee, C., & Terblanche, E. (2017). The effect of three different exercise training modalities on cognitive and physical function in a healthy older population. *European Review of Aging and Physical Activity*, *14*(1), 13. https://doi.org.ezproxy.lib.monash.edu.au/10.1186/s11556-017-0183-5

Cohn, N. B., Dustman, R. E., & Bradford, D. C. (1984). Age‐related decrements in stroop color test performance. *Journal of clinical psychology*, *40*(5), 1244-1250. https://doi-org.ezproxy.lib.monash.edu.au/10.1002/1097-4679(198409)40:5<1244::AID-JCLP2270400521>3.0.CO;2-D

Colcombe, S. J., Kramer, A. F., Erickson, K. I., Scalf, P., McAuley, E., Cohen, N. J., ... & Elavsky, S. (2004). Cardiovascular fitness, cortical plasticity, and aging. *Proceedings of the National Academy of Sciences*, *101*(9), 3316-3321. https://doi-org.ezproxy.lib.monash.edu.au/10.1002/1097-4679(198409)40:5<1244::AID-JCLP2270400521>3.0.CO;2-D

Collette, F., Germain, S., Hogge, M., & Van der Linden, M. (2009). Inhibitory control of memory in normal ageing: Dissociation between impaired intentional and preserved unintentional processes. *Memory*, *17*(1), 104-122. doi: 10.1080/09658210802574146

Congdon, E., Mumford, J. A., Cohen, J. R., Galvan, A., Aron, A. R., Xue, G., ... & Poldrack, R. A. (2010). Engagement of large-scale networks is related to individual differences in inhibitory control. *Neuroimage*, *53*(2), 653-663. doi: 10.1016/j.neuroimage.2010.06.062

Cortese, S., Ferrin, M., Brandeis, D., Buitelaar, J., Daley, D., Dittmann, R. W., ... & Zuddas, A. (2015). Cognitive training for attention-deficit/hyperactivity disorder: meta-analysis of clinical and neuropsychological outcomes from randomized controlled trials. *Journal of the American Academy of Child & Adolescent Psychiatry*, *54*(3), 164-174. https://doi.org/10.1016/j.jaac.2014.12.010

Coxon, J. P., Cash, R. F., Hendrikse, J. J., Rogasch, N. C., Stavrinos, E., Suo, C., & Yücel, M. (2018). GABA concentration in sensorimotor cortex following high‐intensity exercise and relationship to lactate levels. *The Journal of physiology*, *596*(4), 691-702. https://doi-org.ezproxy.lib.monash.edu.au/10.1113/JP274660

Coxon, J. P., Cash, R. F., Hendrikse, J. J., Rogasch, N. C., Stavrinos, E., Suo, C., & Yücel, M. (2018). GABA concentration in sensorimotor cortex following high‐intensity exercise and relationship to lactate levels. *The Journal of physiology*, *596*(4), 691-702. https://doi-org.ezproxy.lib.monash.edu.au/10.1113/JP274660

Den Ouden, L., Kandola, A., Suo, C., Hendrikse, J., Costa, R. J., Watt, M. J., ... & Yücel, M. (2018). The influence of aerobic exercise on hippocampal integrity and function: preliminary findings of a multi-modal imaging analysis. *Brain Plasticity*, *4*(2), 211-216. doi: 10.3233/BPL-170053

Devereux-Fitzgerald, A., Powell, R., Dewhurst, A., & French, D. P. (2016). The acceptability of physical activity interventions to older adults: A systematic review and meta-synthesis. *Social Science & Medicine*, *158*, 14-23. https://doi.org/10.1016/j.socscimed.2016.04.006

Diepeveen, S., Ling, T., Suhrcke, M., Roland, M., & Marteau, T. M. (2013). Public acceptability of government intervention to change health-related behaviours: a systematic review and narrative synthesis. *BMC public health*, *13*(1), 1-11. https://doi-org.ezproxy.lib.monash.edu.au/10.1186/1471-2458-13-756

Ditye, T., Jacobson, L., Walsh, V., & Lavidor, M. (2012). Modulating behavioral inhibition by tDCS combined with cognitive training. *Experimental brain research*, *219*(3), 363-368. doi: 10.1007/s00221-012-3098-4

Dong, C., Minkov, R., Bahar-Fuchs, A., Ellis, K. A., Lautenschlager, N. T., Wade, A. T., ... & Lampit, A. (2020). Combined physical and cognitive training for older adults with and without cognitive impairment: A systematic review and network meta-analysis of randomized controlled trials. doi: 10.1016/j.arr.2020.101232

Eggenberger, P., Wolf, M., Schumann, M., & de Bruin, E. D. (2016). Exergame and balance training modulate prefrontal brain activity during walking and enhance executive function in older adults. *Frontiers in aging neuroscience*, *8*, 66. https://doi.org/10.3389/fnagi.2016.00066

Ehninger, D., & Kempermann, G. (2003). Regional effects of wheel running and environmental enrichment on cell genesis and microglia proliferation in the adult murine neocortex. *Cerebral cortex*, *13*(8), 845-851. https://doi-org.ezproxy.lib.monash.edu.au/10.1093/cercor/13.8.845

Enright, S. J., & Beech, A. R. (1993). Reduced cognitive inhibition in obsessive—compulsive disorder. *British Journal of Clinical Psychology*, *32*(1), 67-74. https://doi-org.ezproxy.lib.monash.edu.au/10.1111/j.2044-8260.1993.tb01028.x

Erickson, K. I., & Kramer, A. F. (2009). Aerobic exercise effects on cognitive and neural plasticity in older adults. *British journal of sports medicine*, *43*(1), 22-24. http://dx.doi.org.ezproxy.lib.monash.edu.au/10.1136/bjsm.2008.052498

Erickson, K. I., Leckie, R. L., & Weinstein, A. M. (2014). Physical activity, fitness, and gray matter volume. *Neurobiology of aging*, *35*, S20-S28. https://doi.org/10.1016/j.neurobiolaging.2014.03.034

Etnier, J. L., Salazar, W., Landers, D. M., Petruzzello, S. J., Han, M., & Nowell, P. (1997). The influence of physical fitness and exercise upon cognitive functioning: A meta-analysis. *Journal of sport and Exercise Psychology*, *19*(3), 249-277. doi: 10.1123/jsep.19.3.249.

Fabel, K., Wolf, S., Ehninger, D., Babu, H., Galicia, P., & Kempermann, G. (2009). Additive effects of physical exercise and environmental enrichment on adult hippocampal neurogenesis in mice. *Frontiers in neuroscience*, *3*, 2. https://doi.org/10.3389/neuro.22.002.2009

Farmer, J., Zhao, X. V., Van Praag, H., Wodtke, K., Gage, F. H., & Christie, B. R. (2004). Effects of voluntary exercise on synaptic plasticity and gene expression in the dentate gyrus of adult male Sprague–Dawley rats in vivo. *Neuroscience*, 124(1). https://doi.org/10.1016/j.neuroscience.2003.09.029

Fiorelli, C. M., Ciolac, E. G., Simieli, L., Silva, F. A., Fernandes, B., Christofoletti, G., & Barbieri, F. A. (2019). Differential acute effect of high-intensity interval or continuous moderate exercise on cognition in individuals with Parkinson’s disease. *Journal of Physical Activity and Health*, *16*(2), 157-164. doi: 10.1123/jpah.2018-0189

Fordyce, D. E., & Farrar, R. P. (1991). Enhancement of spatial learning in F344 rats by physical activity and related learning-associated alterations in hippocampal and cortical cholinergic functioning. *Behavioural brain research,* 46(2), 123-133. https://doi.org/10.1016/S0166-4328(05)80105-6

Freedland, K. E., King, A. C., Ambrosius, W. T., Mayo-Wilson, E., Mohr, D. C., Czajkowski, S. M., ... & Riley, W. T. (2019). The selection of comparators for randomized controlled trials of health-related behavioral interventions: recommendations of an NIH expert panel. *Journal of clinical epidemiology*, 110, 74-81. https://doi.org/10.1016/j.jclinepi.2019.02.011

Gates, N., Singh, M. A. F., Sachdev, P. S., & Valenzuela, M. (2013). The effect of exercise training on cognitive function in older adults with mild cognitive impairment: a meta-analysis of randomized controlled trials. *The American Journal of Geriatric Psychiatry*, *21*(11), 1086-1097. https://doi.org/10.1016/j.jagp.2013.02.018

Geurts, H. M., van den Bergh, S. F., & Ruzzano, L. (2014). Prepotent response inhibition and interference control in autism spectrum disorders: Two meta‐analyses. *Autism Research*, *7*(4), 407-420. https://doi-org.ezproxy.lib.monash.edu.au/10.1002/aur.1369

Gogniat, M. A., Robinson, T. R., & Miller, L. S. (2021). Exercise interventions do not impact brain volume change in older adults: A Systematic review and meta-analysis. *Neurobiology of Aging*. https://doi.org/10.1016/j.neurobiolaging.2021.01.025

Guye, S., De Simoni, C., & von Bastian, C. C. (2017). Do individual differences predict change in cognitive training performance? A latent growth curve modeling approach. *Journal of Cognitive Enhancement*, *1*(4), 374-393. https://doi-org.ezproxy.lib.monash.edu.au/10.1007/s41465-017-0049-9

Haier, R. J., Siegel Jr, B. V., MacLachlan, A., Soderling, E., Lottenberg, S., & Buchsbaum, M. S. (1992). Regional glucose metabolic changes after learning a complex visuospatial/motor task: a positron emission tomographic study. *Brain research*, *570*(1-2), 134-143. https://doi.org/10.1016/0006-8993(92)90573-R

Hedden, T., & Gabrieli, J. D. (2004). Insights into the ageing mind: a view from cognitive neuroscience. *Nature reviews neuroscience*, *5*(2), 87-96.
https://doi-org.ezproxy.lib.monash.edu.au/10.1038/nrn1323

Hempel, A., Giesel, F. L., Garcia Caraballo, N. M., Amann, M., Meyer, H., Wüstenberg, T., ... & Schröder, J. (2004). Plasticity of cortical activation related to working memory during training. *American Journal of Psychiatry*, *161*(4), 745-747. https://doi.org/10.1176/appi.ajp.161.4.745

Hendrikse, J., Kandola, A., Coxon, J., Rogasch, N., & Yücel, M. (2017). Combining aerobic exercise and repetitive transcranial magnetic stimulation to improve brain function in health and disease. *Neuroscience & Biobehavioral Reviews*, *83*, 11-20. https://doi.org/10.1016/j.neubiorev.2017.09.023

Hess, G. R. Z. E. G. O. R. Z., Aizenman, C. D., & Donoghue, J. P. (1996). Conditions for the induction of long-term potentiation in layer II/III horizontal connections of the rat motor cortex. *Journal of neurophysiology*, *75*(5), 1765-1778. https://doi-org.ezproxy.lib.monash.edu.au/10.1152/jn.1996.75.5.1765

Heyn, P., Abreu, B. C., & Ottenbacher, K. J. (2004). The effects of exercise training on elderly persons with cognitive impairment and dementia: a meta-analysis. *Archives of physical medicine and rehabilitation*, *85*(10), 1694-1704. https://doi.org/10.1016/j.apmr.2004.03.019

Higgins, J. P., Thomas, J., Chandler, J., Cumpston, M., Li, T., Page, M. J., & Welch, V. A. (Eds.). (2019). *Cochrane handbook for systematic reviews of interventions*. John Wiley & Sons

Hillman, C. H., Pontifex, M. B., Castelli, D. M., Khan, N. A., Raine, L. B., Scudder, M. R., ... & Kamijo, K. (2014). Effects of the FITKids randomized controlled trial on executive control and brain function. *Pediatrics*, *134*(4), e1063-e1071. https://doi-org.ezproxy.lib.monash.edu.au/10.1542/peds.2013-3219

Hötting, K., & Röder, B. (2013). Beneficial effects of physical exercise on neuroplasticity and cognition. *Neuroscience & Biobehavioral Reviews*, *37*(9), 2243-2257. https://doi.org/10.1016/j.neubiorev.2013.04.005

Hudson, A., & Jacques, S. (2014). Put on a happy face! Inhibitory control and socioemotional knowledge predict emotion regulation in 5-to 7-year-olds. *Journal of Experimental Child Psychology*, *123*, 36-52. https://doi.org/10.1016/j.jecp.2014.01.012

Hwang, J., Brothers, R. M., Castelli, D. M., Glowacki, E. M., Chen, Y. T., Salinas, M. M., ... & Calvert, H. G. (2016). Acute high-intensity exercise-induced cognitive enhancement and brain-derived neurotrophic factor in young, healthy adults. *Neuroscience letters*, *630*, 247-253. https://doi.org/10.1016/j.neulet.2016.07.033

Hyodo, K., Dan, I., Kyutoku, Y., Suwabe, K., Byun, K., Ochi, G., ... & Soya, H. (2016). The association between aerobic fitness and cognitive function in older men mediated by frontal lateralization. *Neuroimage*, *125*, 291-300. https://doi.org/10.1016/j.neuroimage.2015.09.062

Iacono, W. G., Carlson, S. R., & Malone, S. M. (2000). Identifying a multivariate endophenotype for substance use disorders using psychophysiological measures. *International Journal of Psychophysiology*, *38*(1), 81-96. https://doi.org/10.1016/S0167-8760(00)00132-X

Ji, Z., Feng, T., Mei, L., Li, A., & Zhang, C. (2019). Influence of acute combined physical and cognitive exercise on cognitive function: an NIRS study. *PeerJ*, 7, e7418. https://doi.org/10.7717/peerj.7418

Kandola, A., Hendrikse, J., Lucassen, P. J., & Yücel, M. (2016). Aerobic exercise as a tool to improve hippocampal plasticity and function in humans: practical implications for mental health treatment. *Frontiers in Human Neuroscience*, *10*, 373.  https://doi.org/10.3389/fnhum.2016.00373

Karssemeijer, E. E., Aaronson, J. J., Bossers, W. W., Smits, T. T., & Kessels, R. R. (2017). Positive effects of combined cognitive and physical exercise training on cognitive function in older adults with mild cognitive impairment or dementia: A meta-analysis. *Ageing research reviews*, *40*, 75-83. https://doi.org/10.1016/j.arr.2017.09.003

Kempermann, G., Kuhn, H. G., & Gage, F. H. (1997). More hippocampal neurons in adult mice living in an enriched environment. *Nature*, 386(6624), 493-495. https://doi.org/10.1038/386493a0

Kempermann, G., Kuhn, H. G., & Gage, F. H. (1998). Experience-induced neurogenesis in the senescent dentate gyrus. *Journal of Neuroscience*, 18(9), 3206-3212. https://doi.org/10.1523/JNEUROSCI.18-09-03206.1998

Kim, T. W., & Sung, Y. H. (2017). Regular exercise promotes memory function and enhances hippocampal neuroplasticity in experimental autoimmune encephalomyelitis mice. *Neuroscience*, *346*, 173-181. https://doi.org/10.1016/j.neuroscience.2017.01.016

Kim, T. W., Choi, H. H., & Chung, Y. R. (2016). Treadmill exercise alleviates impairment of cognitive function by enhancing hippocampal neuroplasticity in the high-fat diet-induced obese mice. *Journal of Exercise Rehabilitation*, *12*(3), 156. doi: 10.12965/jer.1632644.322

Krafft, C. E., Schwarz, N. F., Chi, L., Weinberger, A. L., Schaeffer, D. J., Pierce, J. E., ... & McDowell, J. E. (2014). An 8‐month randomized controlled exercise trial alters brain activation during cognitive tasks in overweight children. *Obesity*, *22*(1), 232-242. https://doi-org.ezproxy.lib.monash.edu.au/10.1002/oby.20518

Kronenberg, G., Bick-Sander, A., Bunk, E., Wolf, C., Ehninger, D., & Kempermann, G. (2006). Physical exercise prevents age-related decline in precursor cell activity in the mouse dentate gyrus. *Neurobiology of aging,* 27(10), 1505-1513. https://doi.org/10.1016/j.neurobiolaging.2005.09.016

Kühn, S., Lorenz, R. C., Weichenberger, M., Becker, M., Haesner, M., O'Sullivan, J., ... & Gallinat, J. (2017). Taking control! Structural and behavioural plasticity in response to game-based inhibition training in older adults. *NeuroImage*, *156*, 199-206. https://doi.org/10.1016/j.neuroimage.2017.05.026

Lauenroth, A., Ioannidis, A. E., & Teichmann, B. (2016). Influence of combined physical and cognitive training on cognition: a systematic review. *BMC geriatrics*, *16*(1), 1-14. https://doi-org.ezproxy.lib.monash.edu.au/10.1186/s12877-016-0315-1

Lee, Y. C., & Chao, H. F. (2012). The role of active inhibitory control in psychological well-being and mindfulness. *Personality and Individual Differences*, *53*(5), 618-621. https://doi.org/10.1016/j.paid.2012.05.001

Levin, O., Netz, Y., & Ziv, G. (2021). Behavioral and Neurophysiological Aspects of Inhibition—The Effects of Acute Cardiovascular Exercise. *Journal of Clinical Medicine*, *10*(2), 282. https://doi.org/10.3390/jcm10020282

Li, M. Y., Huang, M. M., Li, S. Z., Tao, J., Zheng, G. H., & Chen, L. D. (2017). The effects of aerobic exercise on the structure and function of DMN-related brain regions: a systematic review. *International Journal of Neuroscience*, *127*(7), 634-649. https://doi-org.ezproxy.lib.monash.edu.au/10.1080/00207454.2016.1212855

Lipszyc, J., & Schachar, R. (2010). Inhibitory control and psychopathology: a meta-analysis of studies using the stop signal task. *J Int Neuropsychol Soc, 16*(6), 1064-1076. doi:10.1017/S1355617710000895

Lista, I., & Sorrentino, G. (2010). Biological mechanisms of physical activity in preventing cognitive decline. *Cellular and molecular neurobiology*, 30(4), 493-503. https://doi-org.ezproxy.lib.monash.edu.au/10.1007/s10571-009-9488-x

López-Higes, R., Martín-Aragoneses, M. T., Rubio-Valdehita, S., Delgado-Losada, M. L., Montejo, P., Montenegro, M., ... & López-Sanz, D. (2018). Efficacy of cognitive training in older adults with and without subjective cognitive decline is associated with inhibition efficiency and working memory span, not with cognitive reserve. *Frontiers in aging neuroscience*, *10*, 23. doi: 10.1017/S1355617710000895

Lubman, D. I., Yücel, M., & Pantelis, C. (2004). Addiction, a condition of compulsive behaviour? Neuroimaging and neuropsychological evidence of inhibitory dysregulation. *Addiction*, *99*(12), 1491-1502. https://doi-org.ezproxy.lib.monash.edu.au/10.1111/j.1360-0443.2004.00808.x

Ludyga, S., Gerber, M., Pühse, U., Looser, V. N., & Kamijo, K. (2020). Systematic review and meta-analysis investigating moderators of long-term effects of exercise on cognition in healthy individuals. *Nature Human Behaviour*, 1-10. https://doi-org.ezproxy.lib.monash.edu.au/10.1038/s41562-020-0851-8

Luijten, M., Machielsen, M. W., Veltman, D. J., Hester, R., de Haan, L., & Franken, I. H. (2014). Systematic review of ERP and fMRI studies investigating inhibitory control and error processing in people with substance dependence and behavioural addictions. *Journal of psychiatry & neuroscience*. https://doi-org.ezproxy.lib.monash.edu.au/10.1503/jpn.130052

Mandyam, C. D., Wee, S., Eisch, A. J., Richardson, H. N., & Koob, G. F. (2007). Methamphetamine self-administration and voluntary exercise have opposing effects on medial prefrontal cortex gliogenesis. *Journal of Neuroscience*, *27*(42), 11442-11450. https://doi.org/10.1523/JNEUROSCI.2505-07.2007

Miller, D. I., Taler, V., Davidson, P. S., & Messier, C. (2012). Measuring the impact of exercise on cognitive aging: methodological issues. *Neurobiology of aging*, 33(3), 622-e29. https://doi.org/10.1016/j.neurobiolaging.2011.02.020

Mooney, R. A., Coxon, J. P., Cirillo, J., Glenny, H., Gant, N., & Byblow, W. D. (2016). Acute aerobic exercise modulates primary motor cortex inhibition. *Experimental brain research*, *234*(12), 3669-3676. https://doi-org.ezproxy.lib.monash.edu.au/10.1007/s00221-016-4767-5

Motta‐Teixeira, L. C., Takada, S. H., Machado‐Nils, A. V., Nogueira, M. I., & Xavier, G. F. (2016). Spatial learning and neurogenesis: effects of cessation of wheel running and survival of novel neurons by engagement in cognitive tasks. *Hippocampus*, *26*(6), 794-803. https://doi-org.ezproxy.lib.monash.edu.au/10.1002/hipo.22560

Nielson, K. A., Langenecker, S. A., & Garavan, H. (2002). Differences in the functional neuroanatomy of inhibitory control across the adult life span. *Psychology and aging*, *17*(1), 56. https://doi-org.ezproxy.lib.monash.edu.au/10.1037/0882-7974.17.1.56

Nigg, J. T., Wong, M. M., Martel, M. M., Jester, J. M., Puttler, L. I., Glass, J. M., ... & Zucker, R. A. (2006). Poor response inhibition as a predictor of problem drinking and illicit drug use in adolescents at risk for alcoholism and other substance use disorders. *Journal of the American Academy of Child & Adolescent Psychiatry*, *45*(4), 468-475. https://doi.org/10.1097/01.chi.0000199028.76452.a9

Northey, J. M., Cherbuin, N., Pumpa, K. L., Smee, D. J., & Rattray, B. (2018). Exercise interventions for cognitive function in adults older than 50: a systematic review with meta-analysis. *British Journal of Sports Medicine*, *52*(3), 154-160. doi: 10.1136/bjsports-2016-096587

O’Callaghan, R. M., Ohle, R., & Kelly, A. M. (2007). The effects of forced exercise on hippocampal plasticity in the rat: A comparison of LTP, spatial-and non-spatial learning. *Behavioural brain research*, 176(2), 362-366. https://doi.org/10.1016/j.bbr.2006.10.018

Oberle, E., Schonert-Reichl, K. A., Lawlor, M. S., & Thomson, K. C. (2012). Mindfulness and inhibitory control in early adolescence. *The Journal of Early Adolescence*, *32*(4), 565-588. https://doi-org.ezproxy.lib.monash.edu.au/10.1177/0272431611403741

Obeso, I., Wilkinson, L., Casabona, E., Bringas, M. L., Alvarez, M., Alvarez, L., ... & Jahanshahi, M. (2011). Deficits in inhibitory control and conflict resolution on cognitive and motor tasks in Parkinson’s disease. *Experimental brain research*, *212*(3), 371-384. doi: 10.1007/s00221-011-2736-6

Olesen, P. J., Westerberg, H., & Klingberg, T. (2004). Increased prefrontal and parietal activity after training of working memory. *Nature neuroscience*, *7*(1), 75-79. https://doi-org.ezproxy.lib.monash.edu.au/10.1038/nn1165

Page, L. A., Rubia, K., Deeley, Q., Daly, E., Toal, F., Mataix-Cols, D., ... & Murphy, D. G. (2009). A functional magnetic resonance imaging study of inhibitory control in obsessive-compulsive disorder. *Psychiatry Research: Neuroimaging*, *174*(3), 202-209. https://doi.org/10.1016/j.pscychresns.2009.05.002

Park, H. S., & Kim, T. W. (2017). Paternal physical exercise improves spatial learning ability by enhancing hippocampal neuroplasticity in male pups born from obese maternal rats. *Journal of exercise rehabilitation*, *13*(3), 266. doi: 10.12965/jer.1734998.499

Radley-Crabb, H., Terrill, J., Shavlakadze, T., Tonkin, J., Arthur, P., & Grounds, M. (2012). A single 30 min treadmill exercise session is suitable for ‘proof-of concept studies’ in adult mdx mice: a comparison of the early consequences of two different treadmill protocols. *Neuromuscular Disorders*, *22*(2), 170-182. https://doi.org/10.1016/j.nmd.2011.07.008

Rector, N. A., Richter, M. A., Lerman, B., & Regev, R. (2015). A pilot test of the additive benefits of physical exercise to CBT for OCD. *Cognitive Behaviour Therapy*, *44*(4), 328-340. https://doi.org/10.1016/j.nmd.2011.07.008

Rioult-Pedotti, M. S., Friedman, D., & Donoghue, J. P. (2000). Learning-induced LTP in neocortex. *Science*, *290*(5491), 533-536. doi: 10.1126/science.290.5491.533

Sacco, G., Caillaud, C., Ben Sadoun, G., Robert, P., David, R., & Brisswalter, J. (2016). Exercise Plus Cognitive Performance Over and Above Exercise Alone in Subjects with Mild Cognitive Impairment. *J Alzheimers Dis, 50*(1), 19-25. doi:10.3233/JAD-150194

Sala, G., Aksayli, N. D., Tatlidil, K. S., Tatsumi, T., Gondo, Y., Gobet, F., ... & Verkoeijen, P. (2019). Near and far transfer in cognitive training: A second-order meta-analysis. *Collabra: Psychology*, *5*(1). doi: https://doi-org.ezproxy.lib.monash.edu.au/10.1525/collabra.203

Sanders, L. M., Hortobágyi, T., la Bastide-van Gemert, S., van der Zee, E. A., & van Heuvelen, M. J. (2019). Dose-response relationship between exercise and cognitive function in older adults with and without cognitive impairment: a systematic review and meta-analysis. *PloS one*, *14*(1). https://doi.org/10.1371/journal.pone.0210036

Saucedo Marquez, C. M., Vanaudenaerde, B., Troosters, T., & Wenderoth, N. (2015). High-intensity interval training evokes larger serum BDNF levels compared with intense continuous exercise. *Journal of applied physiology*, *119*(12), 1363-1373. https://doi-org.ezproxy.lib.monash.edu.au/10.1152/japplphysiol.00126.2015

Schachar, R., Mota, V. L., Logan, G. D., Tannock, R., & Klim, P. (2000). Confirmation of an inhibitory control deficit in attention-deficit/hyperactivity disorder. *Journal of abnormal child psychology*, *28*(3), 227-235. https://doi-org.ezproxy.lib.monash.edu.au/10.1023/A:1005140103162

Shatil, E. (2013). Does combined cognitive training and physical activity training enhance cognitive abilities more than either alone? A four-condition randomized controlled trial among healthy older adults. *Front Aging Neurosci, 5*, 8. doi:10.3389/fnagi.2013.00008

Singh, A. M., Duncan, R. E., Neva, J. L., & Staines, W. R. (2014). Aerobic exercise modulates intracortical inhibition and facilitation in a nonexercised upper limb muscle. *BMC sports science, medicine and rehabilitation*, *6*(1), 23. https://doi-org.ezproxy.lib.monash.edu.au/10.1186/2052-1847-6-23

Smith, A. E., Goldsworthy, M. R., Garside, T., Wood, F. M., & Ridding, M. C. (2014). The influence of a single bout of aerobic exercise on short-interval intracortical excitability. *Experimental brain research*, *232*(6), 1875-1882. https://doi-org.ezproxy.lib.monash.edu.au/10.1007/s00221-014-3879-z

Smith, J. L., Mattick, R. P., Jamadar, S. D., & Iredale, J. M. (2014). Deficits in behavioural inhibition in substance abuse and addiction: a meta-analysis. *Drug and alcohol dependence*, *145*, 1-33. https://doi.org/10.1016/j.drugalcdep.2014.08.009

Smith, P. J., Blumenthal, J. A., Hoffman, B. M., Cooper, H., Strauman, T. A., Welsh-Bohmer, K., ... & Sherwood, A. (2010). Aerobic exercise and neurocognitive performance: a meta-analytic review of randomized controlled trials. *Psychosomatic medicine*, *72*(3), 239. doi: 10.1097/PSY.0b013e3181d14633

Sonuga-Barke, E., Brandeis, D., Holtmann, M., & Cortese, S. (2014). Computer-based cognitive training for ADHD: a review of current evidence. *Child and Adolescent Psychiatric Clinics*, *23*(4), 807-824. https://doi.org/10.1016/j.chc.2014.05.009

Staiano, A. E., Abraham, A. A., & Calvert, S. L. (2012). Competitive versus cooperative exergame play for african american adolescents' executive function skills: Short-term effects in a long-term training intervention. *Developmental Psychology, 48*(2), 337-342. doi:10.1037/a0026938

Stavrinos, E. L., & Coxon, J. P. (2017). High-intensity interval exercise promotes motor cortex disinhibition and early motor skill consolidation. *Journal of cognitive neuroscience*, *29*(4), 593-604. https://doi-org.ezproxy.lib.monash.edu.au/10.1162/jocn_a_01078

Stillman, C. M., Cohen, J., Lehman, M. E., & Erickson, K. I. (2016). Mediators of physical activity on neurocognitive function: a review at multiple levels of analysis. *Frontiers in human neuroscience*, *10*, 626. https://doi.org/10.3389/fnhum.2016.00626

Stillman, C. M., Esteban-Cornejo, I., Brown, B., Bender, C. M., & Erickson, K. I. (2020). Effects of exercise on brain and cognition across age groups and health states. *Trends in neurosciences*. https://doi.org/10.1016/j.tins.2020.04.010

Suzuki, T., Shimada, H., Makizako, H., Doi, T., Yoshida, D., Tsutsumimoto, K., ... & Park, H. (2012). Effects of multicomponent exercise on cognitive function in older adults with amnestic mild cognitive impairment: a randomized controlled trial. *BMC neurology*, *12*(1), 128. https://doi-org.ezproxy.lib.monash.edu.au/10.1186/1471-2377-12-128

Swick, D., Ashley, V., & Turken, U. (2011). Are the neural correlates of stopping and not going identical? Quantitative meta-analysis of two response inhibition tasks. *Neuroimage*, *56*(3), 1655-1665. https://doi.org/10.1016/j.neuroimage.2011.02.070

Szuhany, K. L., Bugatti, M., & Otto, M. W. (2015). A meta-analytic review of the effects of exercise on brain-derived neurotrophic factor. *Journal of psychiatric research*, *60*, 56-64. https://doi.org/10.1016/j.jpsychires.2014.10.003

Tsukamoto, H., Suga, T., Takenaka, S., Tanaka, D., Takeuchi, T., Hamaoka, T., ... & Hashimoto, T. (2016). Greater impact of acute high-intensity interval exercise on post-exercise executive function compared to moderate-intensity continuous exercise. *Physiology & behavior*, *155*, 224-230. https://doi.org/10.1016/j.physbeh.2015.12.021

Turton, R., Nazar, B. P., Burgess, E. E., Lawrence, N. S., Cardi, V., Treasure, J., & Hirsch, C. R. (2018). To go or not to go: A proof of concept study testing food‐specific inhibition training for women with eating and weight disorders. *European Eating Disorders Review*, *26*(1), 11-21. https://doi- org.ezproxy.lib.monash.edu.au/10.1002/erv.2566

Van Praag, H. (2008). Neurogenesis and exercise: past and future directions. *Neuromolecular medicine*, *10*(2), 128-140. doi: 10.1007/s12017-008-8028-z

Van Praag, H., Christie, B. R., Sejnowski, T. J., & Gage, F. H. (1999). Running enhances neurogenesis, learning, and long-term potentiation in mice. *Proceedings of the National Academy of Sciences*, 96(23), 13427-13431. https://doi-org.ezproxy.lib.monash.edu.au/10.1073/pnas.96.23.13427

Van Praag, H., Kempermann, G., & Gage, F. H. (1999). Running increases cell proliferation and neurogenesis in the adult mouse dentate gyrus. *Nature neuroscience*, 2(3), 266-270. https://doi.org/10.1038/6368.

Van Velzen, L. S., Vriend, C., de Wit, S. J., & van den Heuvel, O. A. (2014). Response inhibition and interference control in obsessive–compulsive spectrum disorders. *Frontiers in human neuroscience*, *8*, 419. https://doi.org/10.3389/fnhum.2014.00419

Verburgh, L., Königs, M., Scherder, E. J., & Oosterlaan, J. (2014). Physical exercise and executive functions in preadolescent children, adolescents and young adults: a meta-analysis. *British journal of sports medicine*, *48*(12), 973-979. doi: 10.1136/bjsports-2012-091441

Verstynen, T. D., Lynch, B., Miller, D. L., Voss, M. W., Prakash, R. S., Chaddock, L., ... & Erickson, K. I. (2012). Caudate nucleus volume mediates the link between cardiorespiratory fitness and cognitive flexibility in older adults. *Journal of aging research*, *2012*. https://doi.org/10.1155/2012/939285

Wanner, P., Cheng, F. H., & Steib, S. (2020). Effects of acute cardiovascular exercise on motor memory encoding and consolidation: A systematic review with meta-analysis. *Neuroscience & Biobehavioral Reviews*. https://doi.org/10.1016/j.neubiorev.2020.06.018

Ward, N., Paul, E., Watson, P., Cooke, G. E., Hillman, C. H., Cohen, N. J., . . . Barbey, A. K. (2017). Enhanced Learning through Multimodal Training: Evidence from a Comprehensive Cognitive, Physical Fitness, and Neuroscience Intervention. *Sci Rep, 7*(1), 5808. doi:10.1038/s41598-017-06237-5.

Weinstein, A. M., Voss, M. W., Prakash, R. S., Chaddock, L., Szabo, A., White, S. M., ... & Erickson, K. I. (2012). The association between aerobic fitness and executive function is mediated by prefrontal cortex volume. *Brain, behavior, and immunity*, *26*(5), 811-819. https://doi.org/10.1016/j.bbi.2011.11.008

Wilckens, K. A., Stillman, C. M., Waiwood, A. M., Kang, C., Leckie, R. L., Peven, J. C., ... & Erickson, K. I. (2020). Exercise interventions preserve hippocampal volume: A meta‐analysis. *Hippocampus*. https://doi-org.ezproxy.lib.monash.edu.au/10.1002/hipo.23292

Xue, Y., Yang, Y., & Huang, T. (2019). Effects of chronic exercise interventions on executive function among children and adolescents: a systematic review with meta-analysis. *British journal of sports medicine*, *53*(22), 1397-1404. http://dx.doi.org.ezproxy.lib.monash.edu.au/10.1136/bjsports-2018-099825

Yücel, M., & Lubman, D. I. (2007). Neurocognitive and neuroimaging evidence of behavioural dysregulation in human drug addiction: implications for diagnosis, treatment and prevention. *Drug and Alcohol Review*, *26*(1), 33-39. https://doi-org.ezproxy.lib.monash.edu.au/10.1080/09595230601036978

Yücel, M., Lubman, D. I., Solowij, N., & Brewer, W. J. (2007). Understanding drug addiction: a neuropsychological perspective. *Australian & New Zealand Journal of Psychiatry*, *41*(12), 957-968. https://doi-org.ezproxy.lib.monash.edu.au/10.1080/00048670701689444

Zhu, X., Yin, S., Lang, M., He, R., & Li, J. (2016). The more the better? A meta-analysis on effects of combined cognitive and physical intervention on cognition in healthy older adults. *Ageing Research Reviews*, *31*, 67-79. https://doi.org/10.1016/j.arr.2016.07.003

**Figures and Captions**
